# Identification guide to larvae of Caucasian *Epeorus* (*Caucasiron*) (Ephemeroptera, Heptageniidae)

**DOI:** 10.3897/zookeys.986.56276

**Published:** 2020-11-05

**Authors:** Ľuboš Hrivniak, Pavel Sroka, Jindřiška Bojková, Roman J. Godunko

**Affiliations:** 1 Biology Centre of the Czech Academy of Sciences, Institute of Entomology, Branišovská 31, 37005 České Budějovice, Czech Republic Biology Centre of the Czech Academy of Sciences České Budějovice Czech Republic; 2 Faculty of Sciences, University of South Bohemia, Branišovská 31, 37005 České Budějovice, Czech Republic University of South Bohemia České Budějovice Czech Republic; 3 Department of Botany and Zoology, Masaryk University, Kotlářská 2, 61137 Brno, Czech Republic Masaryk University Brno Czech Republic; 4 Department of Invertebrate Zoology and Hydrobiology, University of Łódź, Banacha 12/16, 90237 Łódź, Poland University of Łódź Łódź Poland

**Keywords:** aquatic insects, mayflies, morphology, identification key

## Abstract

The Caucasus and adjacent areas are inhabited by fifteen species of mayflies of the genus *Epeorus*, subgenus Caucasiron Kluge, 1997 (Heptageniidae). This identification guide aims to facilitate an accurate species identification of their larvae and sum up all available information on their taxonomy and distribution. An identification key is provided, and the important diagnostic characters of all species are described and illustrated. The larva of E. (C.) insularis (Braasch, 1983) is described for the first time. This study enables the routine identification of *Caucasiron* larvae necessary for biomonitoring and hydrobiological research in the Caucasus region.

## Introduction

The knowledge facilitating the identification of mayflies inhabiting the Caucasus biodiversity hotspot (Myers et al. 2000) is limited to checklists (e.g., Bojková et al. 2018: Iran; Gabelashvili et al. 2018: Georgia; Hrivniak et al. 2018: Armenia) and alpha taxonomic papers focused mostly on the delimitation of newly described species/taxa. The available identification keys deal with selected genera only (Sinitshenkova 1976, 1979: *Epeorus* Eaton, 1881 and *Rhithrogena* Eaton, 1882, respectively; Jacob and Zimmerman 1978: *Baetis* Leach, 1815) or mayfly fauna of the wider region without sufficient information on Caucasian species specifically (Kluge 1997a). These keys are largely outdated, because the number of species newly described from the Caucasus has been steadily increasing in recent years (e.g., Hrivniak et al. 2017; 2018; 2019; 2020a; Martynov and Godunko 2017; Bojková et al. 2018). Therefore, the identification of larvae to the species level is complicated due to the necessity of compiling information from original descriptions and requires advanced experience with the taxonomy of mayflies and comparative collections. Modern identification keys are needed especially for researchers implementing biomonitoring programmes and routine hydrobiological surveys in the region. They often use data on the generic or family level only (e.g., Hakobyan et al. 2010; Asatryan et al. 2016; Hovhannisyan and Shahnazaryan 2016; Sharifinia et al. 2016) and often include numerous misidentifications (cf. Bojková et al. 2018). This study aims to partly fill this gap by providing a complex identification guide for the larvae of the genus *Epeorus*, subgenus Caucasiron Kluge, 1997 (Heptageniidae) (herineafter *Caucasiron*) occurring in the Caucasus and adjacent areas. The *Epeorus* s. l. larvae are known to be sensitive to pollution, are relatively stenotopic, restricted to lotic habitats, and form an ecologically important component in macroinvertebrate assemblages (Morisi et al. 2003). *Caucasiron* species, together with the remaining representatives of Heptageniidae, can, therefore, be used as indicators in water quality assessments and hydrobiological surveys in the Caucasus region.

**Figure 1.**
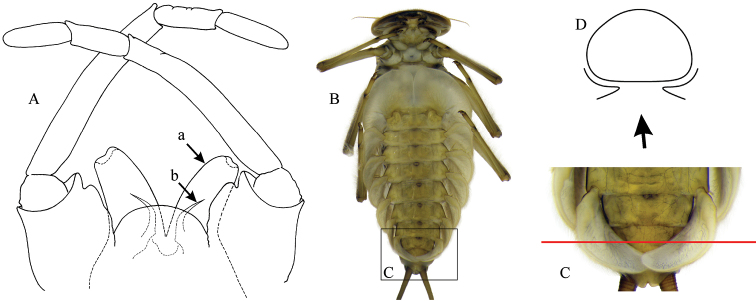
General morphology of Epeorus (Caucasiron): **A** male genitalia (a, penis lobus; b, titillator) **B** larva in ventral view **C** gills VII (in natural position from ventral view) **D** cross section of gills VII showing longitudinal fold.

*Caucasiron* ranks among the most diverse mayfly groups in the Caucasus region, together with the genera *Rhithrogena*, *Electrogena* Zurwerra & Tomka, 1985, and *Ecdyonurus* Eaton, 1868. It is a monophyletic subgenus, sister to the subgenus Iron Eaton, 1885 distributed in North, Central and East Asia and the Nearctic Region (Hrivniak et al. 2020b). Kluge (1997b) defined *Caucasiron* based on the unique apomorphy among Heptageniidae (and mayflies in general), a projection on the costal margin of the gill plates II–VII (see Fig. 5G, arrow). Other morphological characters of *Caucasiron* include: gill plates forming a “suction disc” (i.e., a structure consisting of enlarged gill plate I and overlapping gill plates II–VII, and gill plate VII with a longitudinal fold allowing it to be bent ventrally under the abdominal segments; Fig. 1B–D) and medio-dorsally directed hair-like setae along the anterior margin of the head (Kluge 2015: 346, fig. 178). Imagines are characterized by tubular penis lobes without dorso-lateral denticles and well developed median titillators (Fig. 1A). For the morphological comparison of *Caucasiron* with other related subgenera of the genus *Epeorus* s. l. see Braasch (2006; *Alpiron* Braasch, 2006, *Ironopsis* Traver, 1935) and Kluge (2004; *Iron*).

The global diversity of *Caucasiron* comprises 17 species (Hrivniak et al. 2020b). This identification guide deals with 14 species inhabiting the Caucasus and neighbouring mountain ranges, such as the Zagros and Taurus Mountains, and one species inhabiting Samos Island. The extralimital species E. (C.) guttatus (Braasch, 1979) from Central Asia, E. (C.) extraordinarius Chen et al., 2010 from south-western China, and other Central Asian species presumably belonging to the genus *Caucasiron* (Hrivniak et al. 2017) are not included. All 15 species included in the guide are easily distinguishable based on both morphology and molecular data (Hrivniak et al. 2017, 2019, 2020a, b). Additionally, Hrivniak et al. (2020b) identified seven other distinct lineages based on molecular data only. Most of these lineages likely represent cryptic species or as yet have no formal morphological description. The distribution of possible cryptic lineages is to be found in the guide remarks of the respective morphotypes.

Individual species of *Caucasiron* have different distribution patterns in the Caucasus. Some species are local endemics to the Greater Caucasus, Pontic, Zagros, or Alborz Mountains. Others are widely distributed throughout the Caucasus and the adjacent areas of Anatolia, Cyprus, Iran, and Iraq (Hrivniak et al. 2017, 2019, 2020a, b). Their distribution and diversity patterns can be explained by geological and climatic history, and land development in the region that have significantly affected the diversification of *Caucasiron* in the Caucasus (Hrivniak et al. 2020b).

We aim to provide information necessary for the accurate species identification of *Caucasiron* to the professional public in order to allow the integration of *Caucasiron* into the hydrobiological surveys and biodiversity monitoring in the Caucasus. The main objectives of this study are to (i) form an identification key based on the reliable morphological characters of larvae, (ii) make an inventory of records of all species, and (iii) describe their geographic and altitudinal distribution based on our extensive data and available literature data. *Caucasiron* imagines are not described because of the lack of unambiguously associated specimens. Only information about whether the subimagines or imagines of a given species are described, how they were associated, and who described them, is provided.

## Material and methods

### Sampling

**Figure 2.**
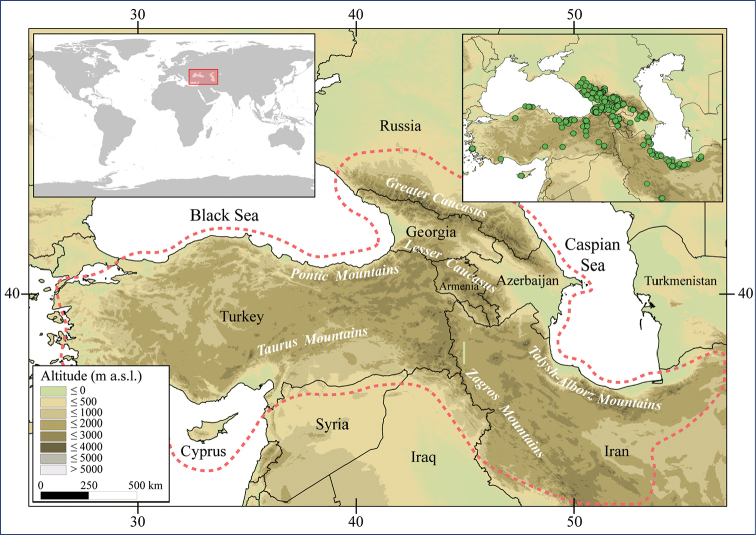
Topographic map of the Caucasus and adjacent mountain ranges with the position of the study area (upper left part) and distribution of our sampling sites (upper right part). Geographical coverage of identification guide of Epeorus (Caucasiron) larvae is defined by red dashed line.

Larvae of *Caucasiron* were collected at 293 localities in Turkey, Georgia, Russia, Armenia, Azerbaijan, Iran, and Samos and Cyprus in 2008–2019 (Fig. 2). They were sampled by a hand net or a metal strainer and fixed in 96% ethanol in the field. Sampling sites fully covered Caucasus region and the geographical distribution of all known Caucasian *Caucasiron* species.

### Morphological examination

Original descriptions of individual species were used for the initial species identification based on morphology (Sinitshenkova 1976; Braasch 1978, 1979, 1980; Braasch and Zimmerman 1979; Braasch and Soldán 1979; Hrivniak et al. 2017, 2019, 2020a). Due to insufficient details given in several of these descriptions, newly collected specimens (both larvae mounted on slides and larvae stored in ethanol) were compared with the type material (holotypes and/or paratypes) to accurately identify the species. Type series were studied in species recently described by us: E. (C.) bicolliculatus Hrivniak, 2017, E. (C.) turcicus Hrivniak, Türkmen & Kazancı, 2019, E. (C.) alborzicus Hrivniak & Sroka, 2020 , E. (C.) shargi Hrivniak & Sroka, 2020, and E. (C.) zagrosicus Hrivniak & Sroka, 2020. Type specimens were also studied in following species: E. (C.) iranicus (Braasch & Soldán, 1979), E. (C.) insularis (Braasch, 1983), E. (C.) magnus (Braasch, 1978), E. (C.) alpestris (Braasch, 1979), E. (C.) soldani (Braasch, 1979), E. (C.) longimaculatus (Braasch, 1980), and E. (C.) sinitshenkovae (Braasch & Zimmerman, 1979). Topotypes were collected and studied in several species: E. (C.) insularis, E. (C.) alpestris, E. (C.) soldani, E. (C.) longimaculatus, E. (C.) iranicus. The extent of morphological variability in each species was mostly determined based on specimens, which species identity was proved by molecular species delimitation (Hrivniak et al. 2017, 2019, 2020a, b).

Body parts with morphological structures requiring microscopical examination (i.e., mouthparts, femora, abdominal terga) were mounted on slide using HydroMatrix® (MicroTech Lab, Graz, Austria) mounting medium. In order to remove the muscle tissue for an investigation of the cuticular structure, the specimens were left overnight in a 10% solution of NaOH prior to slide mounting. Drawings were made using a stereomicroscope Olympus SZX7 and a microscope Olympus BX41, both equipped with a drawing attachment. Photographs were obtained using Leica DFC450 camera fitted with macroscope Leica Z16 APO and folded in Helicon Focus version 5.3. All photographs were subsequently enhanced with Adobe Photoshop™ CS5. The terminology was used mostly according to Kluge and Novikova (2011) and Kluge (2004, 2015).

## Results and discussion

### Larval morphological diagnostic characters

A set of larval diagnostic characters used in the identification guide (listed below) was derived from Braasch and Soldán (1979), who proposed them for the distinguishing larvae of the genus *Iron*. In the concept of Braasch and Soldán (1979), *Iron* included currently recognized taxa *Iron*, *Ironopsis*, *Caucasiron*, and *Alpiron* (see Hrivniak et al. 2020b for the revised concept and phylogeny of these taxa). Individual diagnostic characters are briefly described and figured for all species.

Morphological characters for the larval identification of *Caucasiron*:

**i) coloration of abdominal terga**: shape of medial macula (Fig. 4I, arrow) and length of lateral stripes (extended dorso-posteriorly or not; Figs 13H, I, 16G respectively). Sometimes the medial macula is visible only partly being concealed by a preceding abdominal segment due to the telescoping contraction of the abdomen. The abdomen must be sufficiently extended manually to expose all length of individual segments to recognize the shape of the medial macula.

**ii) coloration of abdominal sterna**: presence/absence and shape of pattern.

The coloration pattern of abdominal terga and/or sterna is often species-specific and valuable in the species identification of *Caucasiron* larvae. It is easily visible and, thus, valuable for the routine identification. However, it often fades in older material or in inadequately fixed larvae, and the intensity of coloration, especially of abdominal sterna, varies among specimens and instars, and may be poorly expressed in some specimens. Therefore, the combination of all characters provided in the guide should be considered for an accurate species identification. The coloration pattern is usually present on terga II–IX (X) and sterna II–VIII (IX). However, patterns vary among segments, therefore, for the purpose of the key we compare terga V–VII and sterna II–VI, which are more species-specific.

**iii) surface of abdominal terga**: presence/absence of outgrowths (protuberances, spines, etc.), shape of sensory setae (hair-like/wide at base; Figs 5E, 20E respectively), density, shape, and sclerotization of denticles along posterior margin of tergum VII (mounting on microscopic slide required).

Except E. (C.) bicolliculatus with a pair of postero-medial protuberances on abdominal terga II–IX (Hrivniak et al. 2017: figs 11, 31, 32; Fig. 34H, arrows), dorsal surface of abdominal terga of Caucasian *Caucasiron* species do not bear any outgrowths or spines.

Denticles along posterior margin of abdominal terga are pointed and irregular in size in all Caucasian *Caucasiron* species. However, the denticles of some species are denser and more sclerotized, e.g., in E. (C.) znojkoi Tshernova, 1938 (Fig. 8E) and E. (C.) nigripilosus (Sinitshenkova, 1976) (Fig. 14E), strongly sclerotized, elongated and curved, e.g., in E. (C.) magnus (Fig. 11E) or less sclerotized and narrowed, e.g., in E. (C.) longimaculatus (Fig. 29E). The pattern of denticles slightly varies among terga and depends on a lateral distance from the midline of a given tergum. Thus, the reference part for the description of denticulation along the posterior margin of terga is used in the key. It is represented by tergum VII, the section from its midline to approximately half distance to the lateral margin.

**iv) medial hypodermal femur spots**: presence/absence and shape (rounded/elongated). The character is relatively stable and usually present on dorsal surface of femora of all leg pairs. Variability was observed in E. (C.) caucasicus (Fig. 4F–H) and includes absence on all or at least some of the legs.

**v) mouthparts**: setation on dorsal surface of labrum (sparse hair-like setae/dense bristle-like setae; Figs 5A, 11A respectively) and shape of mandibular incisors (blunt/pointed) (mounting on microscopic slide required).

Mouthparts of Caucasian *Caucasiron* species are generally without distinct diagnostic characters in most of the species. The only exception is labrum, mandibles and maxillae of E. (C.) magnus. This species differs from all others by setation of dorsal surface of labrum (dense bristle-like setae; Fig. 11A), pointed mandibular incisors (Fig. 11B, C), and thickened maxillary dentisetae (not figured). The shape of labrum is generally variable in most of the Caucasian *Caucasiron*. Exceptions are E. (C.) magnus, E. (C.) alpestris, and E. (C.) sinitshenkovae, the shape of labrum of which can be considered as one of the diagnostic characters. However, it should be noted that the shape of labrum is often distorted during the slide preparation and should be observed in natural position (not flattened), as well as suggested for other Heptageniidae (e.g., *Ecdyonurus*) (Bauernfeind 1997). Therefore, the shape of labra figured in the guide are not flattened on slide but drawn from dorsal view in natural position. Drawings of the shape of mandibular incisors were based on flattened incisors on slides. Despite mandibular incisors are not considered as distinct character in the species identification, they are figured in the guide for comparison with E. (C.) magnus, and for purposes of further taxonomy, in case some new species with different incisors will be found in the future.

**vi) gill plates**: size of a projection on costal margin of gill plates III (with/without distinct projection; Fig. 5G, arrow and Fig. 29G respectively) and shape of gill plates VII in natural position from ventral view (narrow/wide).

The shape of gill plates I–VI is more or less identical between individual species. However, the gill plate VII is specific for some species; e.g., narrow, banana-shaped plate in E. (C.) soldani (Figs 19L, 20H–K), E. (C.) longimaculatus (Figs 28L, M, 29H–L), or E. (C.) sinitshenkovae (Figs 25J; 26H–K); wider and rounded shape in E. (C.) nigripilosus (Figs 13K, 14H–J) or E. (C.) alborzicus (Figs 40J, K, 41H–J). Importantly, the shape of the gill plate VII must be observed in natural position from ventral view, without flattening on a slide (as shown e.g., in Fig. 7L–P). As a part of the gill plate VII is longitudinally bent under the abdomen (Fig. 1C, D), its shape is often deformed during the slide preparation by straightening of its inner margin.

**vii) tarsal claw denticulation**: number of denticles.

Denticulation of tarsal claws was omitted in the guide, due to its high overlap among species and frequent abrasion. Tarsal claws of all species usually possess 2–4 denticles.

**viii) shape of head in fully grown larvae**: ellipsoid/oval trapezoidal/sharply trapezoidal.

The shape of head (in dorsal view) may be used as one of the diagnostic characters in some species; e.g., E. (C.) znojkoi is characteristic by a distinctly angular, sharply trapezoidal head (Fig. 7D), E. (C.) magnus and E. (C.) shargi by more oval trapezoidal head with more broadly rounded corners (Fig. 10D, E and Fig. 43D respectively), and E. (C.) longimaculatus by more or less rounded, ellipsoid shape of head (Fig. 28D). Interspecific differences in the shape of head are most distinct in fully grown or late instars of males (and females in E. (C.) magnus).

**ix) postero-lateral projection on tergum X**: presence/absence (Fig. 11K–M, arrows and 17L respectively) and its size.

We also figure a shape of medial emargination of female sternum IX and spatulate setae on dorsal surface of femora (figures in the guide include the variability from proximal to distal margin of femora of all leg pairs). Despite a relatively wide range of variability in these characters, it may be helpful in identification of some species.

### Identification guide to larvae of Caucasian species of *Caucasiron*

#### How to use the guide?

The dichotomous key divides *Caucasiron* species into two morphological groups, further divided into subgroups. They do not correspond with the phylogeny and merely represent groupings defined for the practical purpose of species identification.

Some characters within the key are subject to variation in some species. For instance, E. (C.) caucasicus usually has a median hypodermal femur spot, but in rare instances it is absent. We deal with this ambiguity by placing such species both in Group A (femur spot present) and B (femur spot absent). Thus, there are sometimes multiple paths leading to the same species in the key.

Most *Caucasiron* species are defined on the basis of a particular combination of several morphological characters. Following species identification using the dichotomous key, it is recommended to compare all the remaining diagnostic characters for a given species, provided in detail in the “Main morphological diagnostic characters of larvae” for each species. Variability of morphological diagnostic characters is described in the remarks section.

The “Main morphological diagnostic characters of larvae” were described based on late-instar larvae (fully-grown larvae). The order of characters is not concise in relation to all species; it always starts with the most prominent character for a given species after which the value of subsequent characters for species identification diminishes. For each species included in the guide, geographical and altitudinal distribution with frequency of sampling sites is provided. The construction of distribution maps was based on published records (Table 1) and our unpublished data. Brief information on distribution is also given directly in the key. Abbreviations correspond with points of the compass; central Greater Caucasus refers to area from Mount Elbrus to Mount Kazbek. In the description of habitat, altitudinal distribution is divided into three categories: low (up to 500 m a.s.l.), middle (500–1500 m), and high (above 1500 m). This serves only for the purpose of rough orientation, since actual environmental conditions on a given altitude may vary significantly because of high climatic heterogeneity within the region. The list of synonyms given for each species includes all generic/subgeneric combinations under which the species is mentioned in the literature, always with the reference to the first study using a given combination.

**Table 1.** Records of *Caucasiron* species from the Caucasus and adjacent areas. Abbreviations used: A-Armenia; N-Nakhchivan; Te-eastern Turkey; T-Turkey*; G-Georgia; AZ-Azerbadijan; I-Iran; Iq-Iraq; Is-Israel; S-Syria; Rw-Russia (western Caucasus); Rc-Russia (central Caucasus); Sa-Samos Island; C-Cyprus Island.

**Species**	**Records and references**
E. (C.) caucasicus (Tshernova, 1938)	**N**-Tshernova (1938); **Rw**,**A**,**G**,**N**-Sinitshenkova (1976), Palatov and Sokolova (2018); **AZ**-Sinitshenkova (1976); **Rc**-Cherchesova (2004); **Te**-Braasch (1981), Koch (1988), Kazancı (2001); **T**-Kazancı and Türkmen (2012)*, Türkmen and Kazancı (2015)
E. (C.) znojkoi (Tshernova, 1938)	**N**,**AZ**-Tshernova (1938); **G,Rc**-Sinitshenkova (1976), Braasch (1980), Cherchesova (2004), Khazeeva (2010); **A**-Sinitshenkova (1976); **Te**-Braasch (1981), Türkmen and Kazancı (2015), Aydınlı (2017); **T**-Kazancı and Türkmen (2012)*; **I**-Bojková et al. (2018)
E. (C.) nigripilosus (Sinitshenkova, 1976)	**G**-Sinitshenkova (1976); **Rc**-Sinitshenkova (1976), Braasch (1979), Khazeeva (2010); **Rw**-Braasch (1979); **Iq**-Al-Zubaidi et al. (1987); **Te**-Kazancı (2001); **T**-Kazancı and Türkmen (2012)*; **I**-Hrivniak et al. (2020a, b); **C**-Hrivniak et al. (2020a, b)
E. (C.) magnus (Braasch, 1978)	**Rw**-Braasch (1978, 1980), Palatov and Sokolova (2018); **G**-Braasch (1980); **A**-Braasch (1980); **T**-Kazancı and Türkmen (2012)*, **Rc**-Cherchesova (2004)
E. (C.) alpestris (Braasch, 1979)	**Rw**; **Rc**-Braasch (1979), Palatov and Sokolova (2018); **Te**-Kazancı (1986, 2001)**; **T**-Kazancı and Türkmen (2012)*, Aydınlı (2017)**,
E. (C.) soldani (Braasch, 1979)	**Rw**; **Rc**-Braasch (1979)
E. (C.) sinitshenkovae (Braasch & Zimmerman, 1979)	**Rc**; **Rw**; **G**-Braasch and Zimmermann (1979)
E. (C.) longimaculatus (Braasch, 1980)	**G**-Braasch (1980), Martynov et al. (2016)**; **Te**-Kazancı and Braasch (1988)**, Kazancı (2001)**; **T**-Kazanci and Turkmen (2012)**
E. (C.) iranicus (Braasch & Soldán, 1979)	**I**-Braasch and Soldán (1979), Mousavi and Hakobyan (2017), Bojková et al. (2018), Hrivniak et al. (2020a, b)
E. (C.) insularis (Braasch, 1983)	**Sa**-Braasch (1983), Hrivniak et al. (2020a, b)
E. (C.) bicolliculatus Hrivniak 2017	**G**-Martynov et al. (2016), Hrivniak et al. (2017); **Te**-Türkmen and Kazancı (2015), Hrivniak et al. (2017); **A**-Švihla (1975)***
E. (C.) turcicus Hrivniak, Türkmen & Kazancı, 2019	**Te**-Hrivniak et al. (2019)
E. (C.) alborzicus Hrivniak & Sroka, 2020	**I**-Hrivniak et al. (2020a)
E. (C.) shargi Hrivniak & Sroka, 2020	**I**-Hrivniak et al. (2020a)
E. (C.) zagrosicus Hrivniak & Sroka, 2020	**I**-Hrivniak et al. (2020a)

* without exact localisation, not included in distribution maps. ** doubtful record not included in distribution maps. *** unpublished record included in the distribution map.

### Key to species

**Table:** 

1	Medial hypodermal femur spots present (e.g., Fig. 13F, G)	**group A**
–	Coloration pattern on abdominal sterna present (Figs 4B; 13B; 46B)	**subgroup A1**, p. 9
–	Coloration pattern on abdominal sterna absent (Figs 28B; 37B; 43B)	**subgroup A2**, p. 9
2	Medial hypodermal femur spots absent (e.g., Fig. 16F)	**group B**
–	Coloration pattern on abdominal sterna present (Figs 4B; 7B; 16B; 31B; 34B; 40B; 19J–K)	**subgroup B1**, p. 10
–	Coloration pattern on abdominal sterna absent (e.g., Figs 10B; 25B)	**subgroup B2**, p. 11
**subgroup A1**		
1	Abdominal sterna II–VI with a pair of oblique stripes (Figs 4J; 22I, J; 46I)	**2**
–	Abdominal sterna II–V (VI) with a pair of triangular spots (Fig. 13J) and abdominal terga with lateral stripes extended dorso-posteriorly (Fig. 13H, I, arrows)	**E. (C.) nigripilosus (W and Central Greater Caucasus, Turkey, Iraq, N Iran)**, p. 19
2	Stripes on abdominal sterna II–VI widened anteriorly (Fig. 46I, arrows) and abdominal terga with lateral stripes extended dorso-posteriorly (Fig. 46H, arrows)	**E. (C.) zagrosicus (S and SW Iran)**, p. 45
–	Stripes on abdominal sterna II–VI not widened anteriorly (Figs 4J; 22I, J)	**3**
3	Abdominal terga V–VII with crown-like medial macula (Fig. 4I)	**E. (C.) caucasicus (widespread in the Caucasus)**, p. 11
–	Abdominal terga V–VII with stripe-like medial macula and a pair of distinct antero-lateral stripes (Fig. 22G, arrows)	**E. (C.) iranicus (N Iran)**, p. 29
**subgroup A2**		
1	Medial hypodermal femur spots distinctly elongated (Fig. 28F–H); setae on abdominal terga wide at base and denticles along posterior margin of tergum VII narrow (Fig. 29E); gill plates III without distinct projection (Fig. 29G); gill plates VII narrow (Figs 28L, M; 29H–L)	**E. (C.) longimaculatus (central Greater Caucasus)**, p. 32
–	Medial hypodermal femur spots rounded, not distinctly elongated, gill plates III with well-developed projection; setae on abdominal terga hair-like (e.g., Fig. 38E)	**2**
2	Abdominal terga V–VII with stripe-like medial macula and lateral stripes extended dorso-posteriorly (Fig. 37G, arrows); gill plates VII narrow (Figs 37I; 38H–K)	**E. (C.) turcicus (NE Turkey, Georgia)**, p. 40
–	Abdominal terga V–VII with more or less triangular or T-shaped medial macula, lateral stripes not extended dorso-posteriorly (Fig. 43I–K); gill plates VII wide (Figs 43M; 44H, I)	**E. (C.) shargi (N Iran)**, p. 45
**subgroup B1**		
1	Setae on abdominal terga wide at base	**2**
–	Setae on abdominal terga hair-like	**3**
2	Abdominal terga II–IX with a pair of postero-medial protuberances (Fig. 34H, arrows; protuberances are most developed on terga VI–VIII and best visible from dorso-lateral view); abdominal terga V–VII with stripe-like medial macula, which is often anteriorly and posteriorly widened (Fig. 34G, H); abdominal sterna II–VI as on Fig. 34J–L	**E. (C.) bicolliculatus (NE Turkey, W Caucasus, Armenia)**, p. 36
–	Abdominal terga without postero-medial protuberances; terga V–VII with well-defined triangular maculae (Fig. 19H, I); sterna not intensively pigmented, pattern of sterna II–VI as on Fig. 19J, K	**E. (C.) soldani (W and central Greater Caucasus)**, p. 24
3	Postero-lateral projections on tergum X distinct (Fig. 41K, arrow); abdominal sterna II–VI with circular medial macula (Fig. 40L–N); gill plates VII wide (Figs 40J, K; 41H–J)	**E. (C.) alborzicus (N Iran)**, p. 41
–	Postero-lateral projections on tergum X absent or indistinct, coloration pattern of abdominal sterna different	**4**
4	Abdominal sterna II–VI yellowish, with a pair of black oblique stripes or brownish rounded medial macula	**5**
–	All or at least abdominal sterna VIII–IX intensively red (Fig. 7L), with reddish to brownish maculation (Fig. 7M) including a longitudinal stripe (Figs 7N–P; 31J) and a pair of reddish oblique (Fig. 7K, a) and/or medio-lateral stripes (Fig. 7K, b)	**6**
5	Abdominal sterna II–VI with a pair of black oblique stripes (Fig. 4J); abdominal terga V–VII with crown-like medial macula (Fig. 4I)	**E. (C.) caucasicus (widespread in the Caucasus, E Turkey)**, p. 11
–	Abdominal sterna II–VI with brownish rounded medial macula (Fig. 16I); abdominal terga V–VII with narrow stripe-like medial macula (widened on terga VIII–IX, Fig. 16G, H, arrows)	**E. (C.) alpestris (W and central Greater Caucasus)**, p. 23
6	Gill plate VII wide (Figs 7J, L–P; 8H–L); denticles along posterior margin of tergum VII relatively long, strongly sclerotized and dense (Fig. 8E); postero-lateral projections on tergum X present or absent (Fig. 8M, N)	**E. (C.) znojkoi s. l. (widespread in the Caucasus, Turkey)**, p. 13
–	Gill plates VII narrow (Figs 31K; 32H, I); denticles along posterior margin of tergum VII relatively short and poorly sclerotized (Fig. 32E); postero-lateral projections on tergum X absent (Fig. 32J)	**E. (C.) insularis (Samos Island, Greece)**, p. 35
**subgroup B2**		
1	Postero-lateral projections on tergum X present (Fig. 11K–M); dorsal surface of labrum with dense bristle-like setae (Fig. 11A); gill plates VII wide or slightly narrowed (Figs 10K; 11H–J)	**E. (C.) magnus (widespread in the Caucasus, Turkey)**, p. 18
–	Postero-lateral projections on tergum X absent (Figs 20L, 26L); gill plates VII distinctly narrowed (Figs 19L; 20H–K; 25J; 26H–K); dorsal surface of labrum with sparse hair-like setae (Figs 20A; 26A)	**2**
2	Abdominal terga V–VII with narrowed triangular medial macula and a pair of anterolateral spots (Fig. 25H; arrows); gill plates III without distinct projection (Fig. 26G); setae on terga not distinctly widened at base, often elongated (Fig. 26E)	**E. (C.) sinitshenkovae (W and central Greater Caucasus)**, p. 30
–	Abdominal terga V–VII with well-defined triangular medial maculae, without a pair of anterolateral spots (Fig. 19H, I); setae on terga wide at base (Fig. 20E); gill plates III with well-developed projection (Fig. 20G)	**E. (C.) soldani (W and central Greater Caucasus)**, p. 24

### Morphological diagnostics, distribution, and habitat of individual species

#### Epeorus (Caucasiron) caucasicus

Taxon classificationAnimaliaEphemeropteraHeptageniidae

(Tshernova, 1938)

7E0EE503-21DC-53E9-BE6C-AC17CDE6DB30

Figs 3 , 4 , 5

Cynigma caucasica Tshernova, 1938
Epeorus (Iron) (Tshernova, 1938); in Tshernova (1974)
Iron fuscus Sinitshenkova, 1976; jun. syn.; in Braasch (1979)
Epeorus (Caucasiron) caucasicus (Tshernova, 1938); in Kluge (1997b)

##### Type locality.

Azerbaijan, The Nakhchivan Autonomous Republic, a stream in the vicinity of the upper Sakarsu River (3000 m a.s.l.).

##### Distribution.

**Figure 3.**
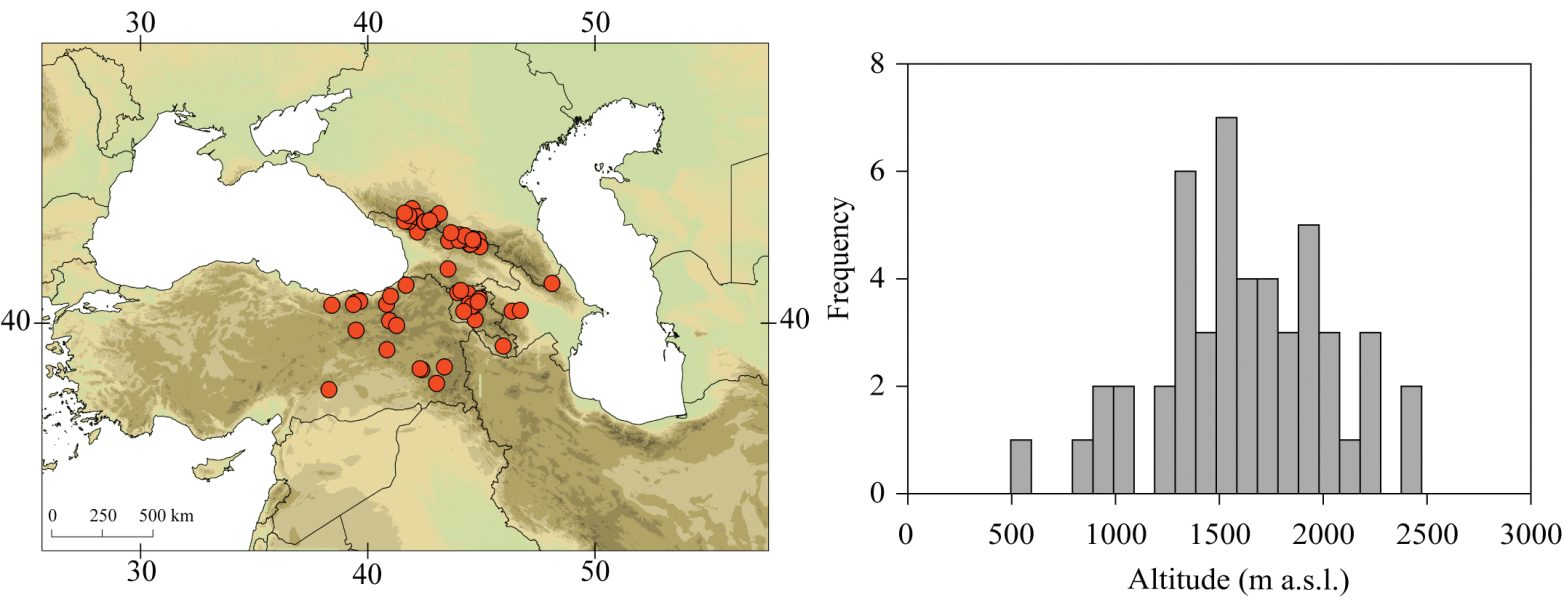
Geographical (left) and vertical (right) distribution of Epeorus (Caucasiron) caucasicus.

Georgia, south-western Russia, Azerbaijan, Armenia, eastern Turkey (Fig. 3). One of the most widespread species in the Caucasus.

##### Habitat.

Larvae inhabit small streams and rivers at middle and high altitude, most frequently found above 1000 m a.s.l. Altitudinal range of sampling sites 496–2474 m a.s.l. (Fig. 3).

##### Main morphological diagnostics of larvae.

**Figure 4.**
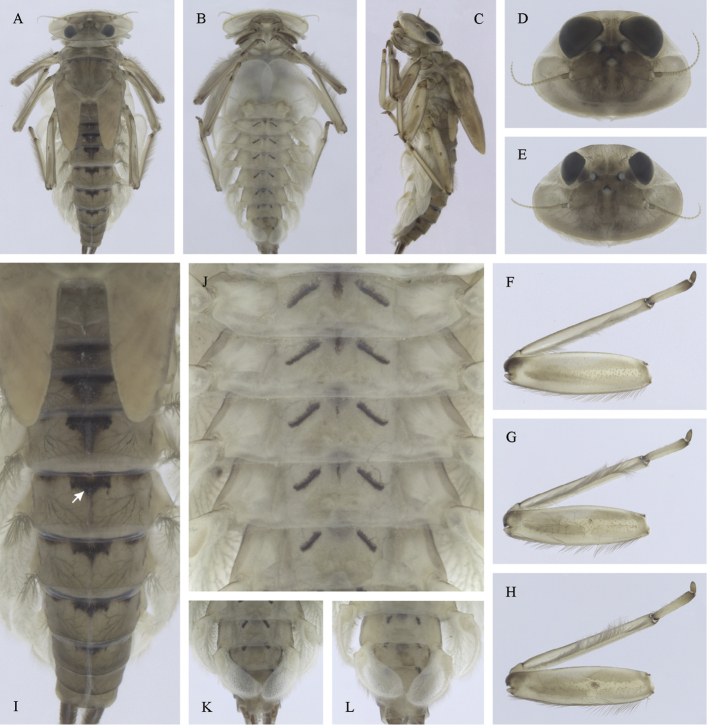
Epeorus (Caucasiron) caucasicus, larva: **A** habitus in dorsal view **B** habitus in ventral view **C** habitus in lateral view **D** head of male in dorsal view **E** head of female in dorsal view **F–H** middle leg in dorsal view **I** abdominal terga (arrow points on medial macula) **J** abdominal sterna II–VI **K, L** gills VII (in natural position from ventral view).

**Figure 5.**
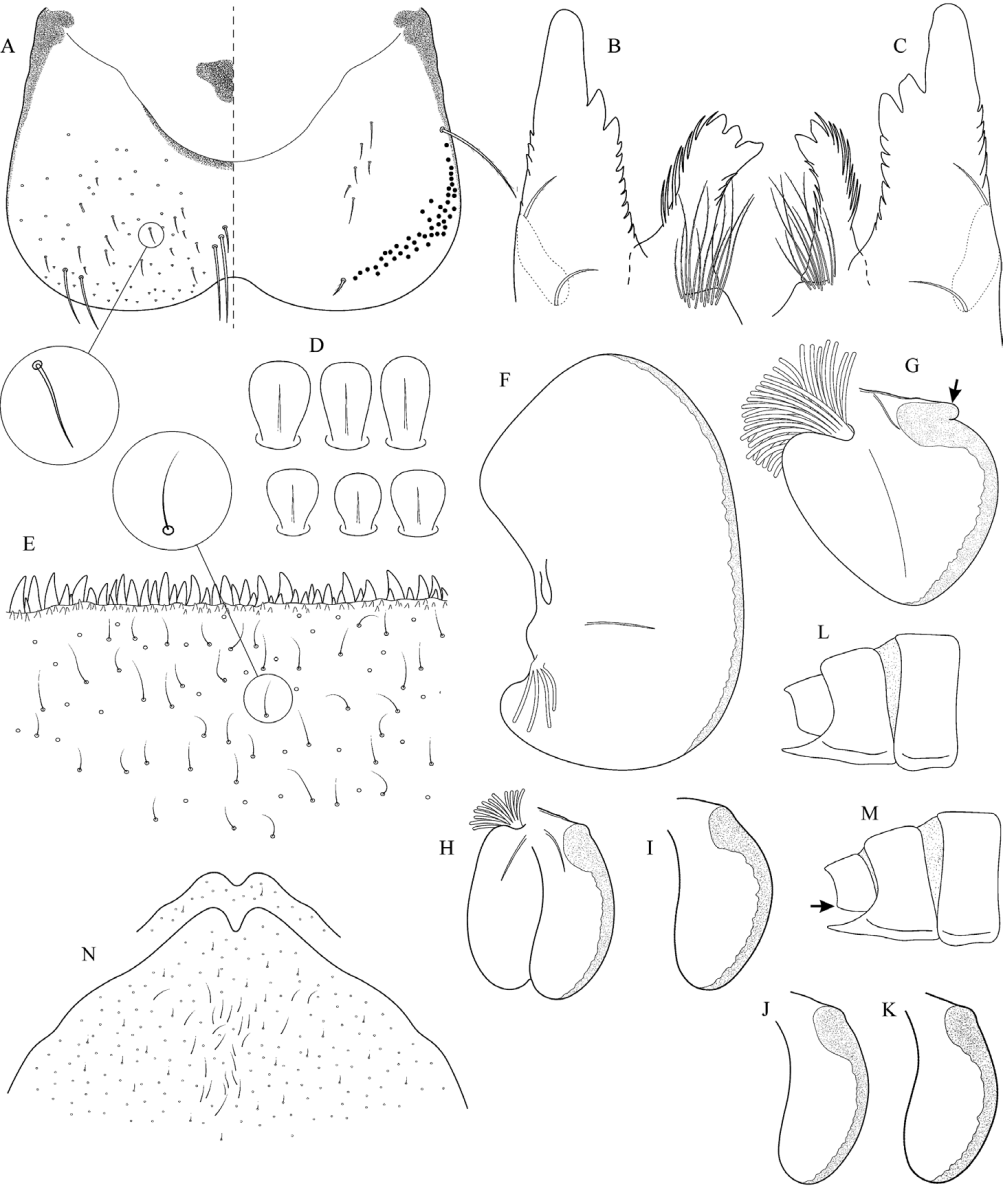
Epeorus (Caucasiron) caucasicus, larva: **A** labrum (left half in dorsal view, right half in ventral view) with detail of hair-like seta **B** incisors of left mandible **C** incisors of right mandible **D** setae on dorsal surface of femora **E** surface and posterior margin of abdominal tergum VII with detail of hair-like seta **F** gill I **G** gill III (arrow points on distinct projection on costal margin) **H** gill VII (flattened on slide) **I–K** gill VII (in natural position from ventral view), variability in shape **L, M** abdominal segments VIII–X in lateral view (arrow points on postero-lateral projection) **N** sternum IX of female with observed variability.

(i) abdominal sterna II–VI with a pair of oblique stripes; nerve ganglia often with stripes or spots (Fig. 4B, J); (ii) abdominal terga V–VII with crown-like medial macula (Fig. 4A, I, arrow); (iii) femora with medial hypodermal spot (Fig. 4G, H), sporadically absent or poorly visible (Fig. 4F); (iv) setae on abdominal terga hair-like (Fig. 5E); (v) gill plates III with well-developed projection (Fig. 5G); (vi) tergum X with poorly developed postero-lateral projections (Fig. 5M, arrow) or without postero-lateral projections (Fig. 5L).

##### Remarks.

***Morphology*.** Coloration pattern of abdominal sterna as in E. (C.) iranicus (Figs 22I, J), similar pattern in E. (C.) zagrosicus (Fig. 46I). Lateral stripes on abdominal terga sporadically dorso-posteriorly extended as in E. (C.) nigripilosus (Fig. 13H, I, arrows). A projection on gill plates III usually well-developed, a slight reduction observed in specimens collected from central Armenia.

***Taxonomy*.** This species was described based on male imagines from the Nakchivan Autonomous Republic (upper Sakarsu River) (Tshernova 1938). The type series is deposited in the Institute of Zoology, Russian Academy of Sciences, Saint Petersburg (IZ) (Kluge 1995). Female imago not described; the larva described by Sinitshenkova (1976) from several localities in Russia, Armenia, and Azerbaijan. Larvae and imagines were associated based on the same sampling sites (a part of the larval material originated from the vicinity of the type locality) and a similarity in the coloration of abdomen of the larva and imagines. The description and validity of larval diagnostic characters were discussed by Braasch (1979, 1980). According to him, Sinitshenkova (1976) described the larva of E. (C.) znojkoi under the name E. (C.) caucasicus by mistake. This opinion was supported by the investigation of imagines reared from larvae corresponding to E. (C.) caucasicus described by Sinitshenkova (1976). Imagines corresponded to E. (C.) znojkoi as were described by Tshernova (1938). The larva belonging to E. (C.) caucasicus was also described in Sinitshenkova (1976), but under erroneous attribution to newly proposed species E. (C.) fuscus. Later, E. (C.) fuscus was considered as a synonym of E. (C.) caucasicus (Braasch 1979; Braasch and Soldán 1979).

#### Epeorus (Caucasiron) znojkoi

Taxon classificationAnimaliaEphemeropteraHeptageniidae

(Tshernova, 1938), sensu lato

DF62BAFE-06B4-5B61-B5DF-B04A171B6386

Figs 6 , 7 , 8

Iron znojkoi Tshernova, 1938
Epeorus (Iron) znojkoi (Tshernova, 1938); in Tshernova (1974)
Iron caucasicus (Tshernova, 1938); in Sinitshenkova (1976) partim
Iron znojkoi Tshernova, 1938; in Sinitshenkova (1976) partim
Epeorus (Caucasiron) znojkoi (Tshernova, 1938); in Kluge (1997b)

##### Type locality.

Azerbaijan, Nakchivan Autonomous Republic, Giljan-tshaj (Gilljak) (2000–2100 m a.s.l.).

##### Distribution.

**Figure 6.**
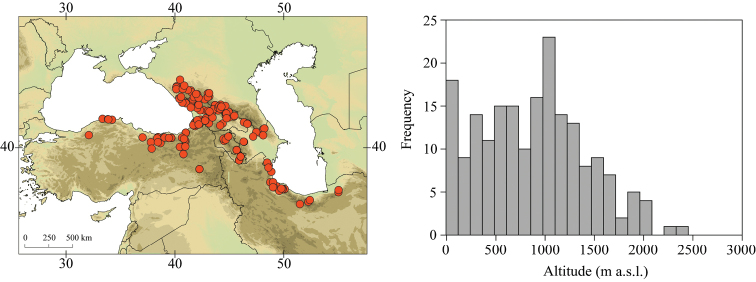
Geographical (left) and vertical (right) distribution of Epeorus (Caucasiron) znojkoi.

Georgia, south-western Russia, Azerbaijan, Armenia, Turkey, northern Iran (Fig. 6). The most widespread species in the Caucasus.

##### Habitat.

Larvae inhabit streams and rivers of various sizes, from larger braided low altitude rivers to small streams at high altitude. Altitudinal range of sampling sites -6–2453 m a.s.l. (Fig. 6). Most frequently found in low and middle altitudes. Often syntopic with E. (C.) magnus.

##### Main morphological diagnostics of larvae.

**Figure 7.**
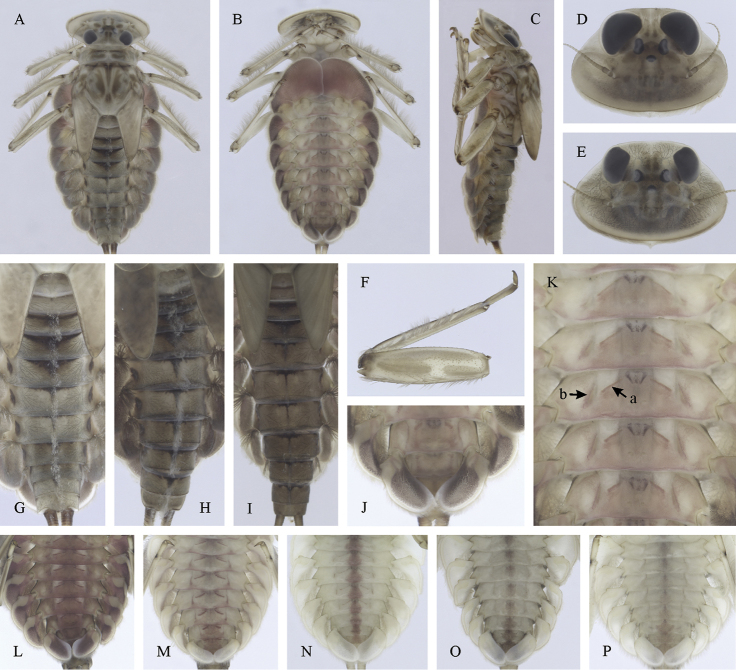
Epeorus (Caucasiron) znojkoi, larva: **A** habitus in dorsal view **B** habitus in ventral view **C** habitus in lateral view **D** head of male in dorsal view **E** head of female in dorsal view **F** middle leg in dorsal view **G–I** abdominal terga **J** gills VII (in natural position from ventral view) **K** abdominal sterna II–VI (a, position of oblique stripes b, position of medio-lateral stripes) **L–P** abdominal sterna, variability in coloration pattern (**L** Georgia **M, O, P** Iran **N** Turkey).

**Figure 8.**
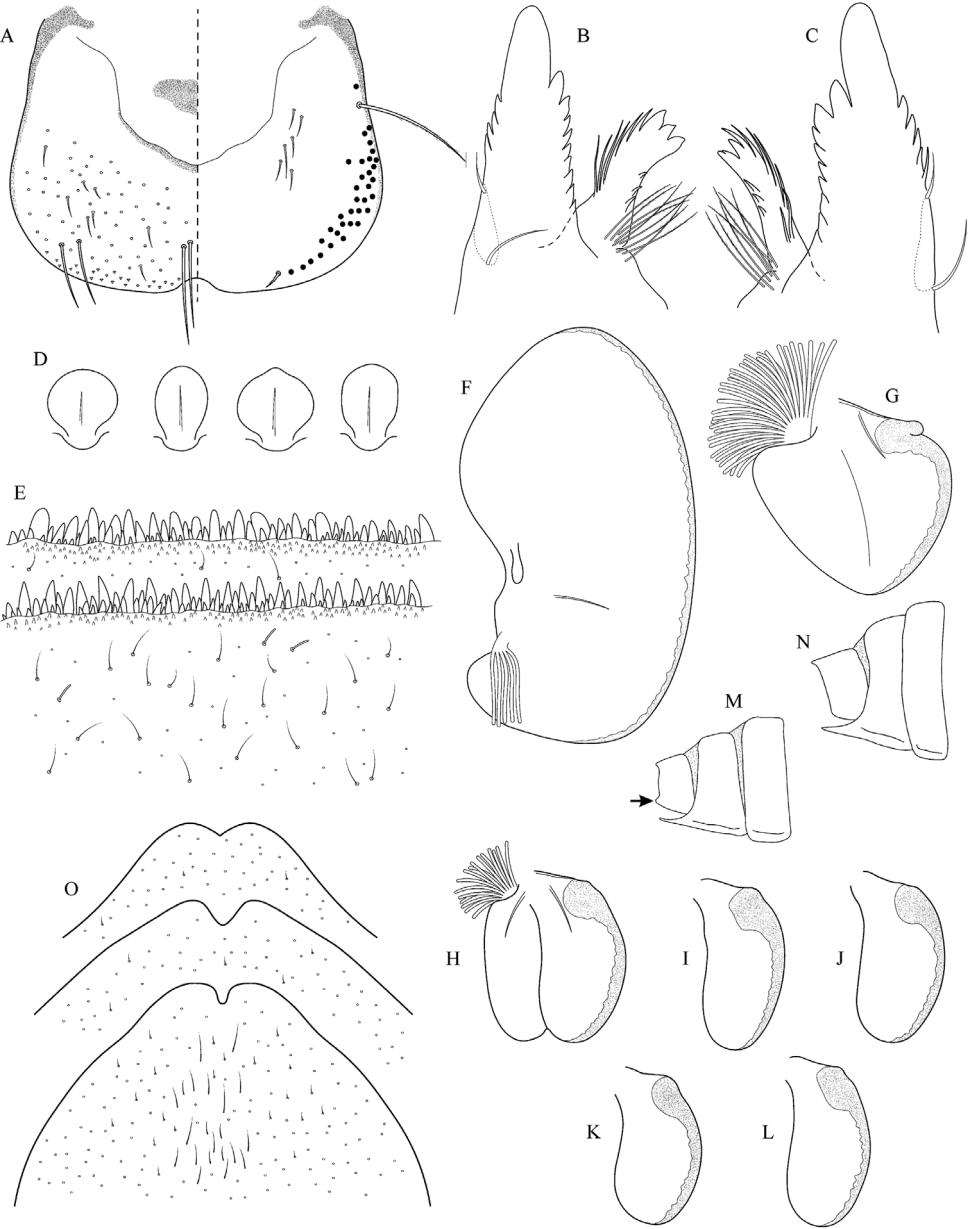
Epeorus (Caucasiron) znojkoi, larva: **A** labrum (left half in dorsal view, right half in ventral view) **B** incisors of left mandible **C** incisors of right mandible **D** setae on dorsal surface of femora **E** surface and posterior margin of abdominal tergum VII and its variability **F** gill I **G** gill III **H** gill VII (flattened on slide) **I–L** gill VII (in natural position from ventral view), variability in shape **M, N** abdominal segments VIII–X in lateral view (arrow points on postero-lateral projection) **O** sternum IX of female with observed variability.

(i) abdominal terga II–IV with triangular medial macula and terga V–VII with T shaped medial macula (Fig. 7A, G–I); (ii) abdominal sterna intensively red or reddish (Fig. 7B, L, M), with a pair of reddish oblique stripes (Fig. 7K, a) and/or reddish medio-lateral stripes (Fig. 7K, b), or with reddish to brownish longitudinal stripe on all sterna or at least on sterna VIII and IX (Fig. 7N–P) (iii); tergum X with short postero-lateral projections (Fig. 8M, arrow) or without postero-lateral projections (Fig. 8N); (iv) femora without medial hypodermal spot (Fig. 7F); (v) gill plates VII (in natural position from ventral view) wide (Figs 7J, L–P, 8H–L); (vi) denticles along posterior margin of tergum VII strongly sclerotized and dense (Fig. 8E); (vii) gill plates III with well-developed projection (Fig. 8G); (viii) shape of head sharply trapezoidal in males (Fig. 7D).

##### Remarks.

***Morphology.*** The reduction of reddish coloration of abdominal sterna observed particularly in specimens collected from Turkey (Fig. 7N) and northern Iran (Fig. 7O, P). Similar coloration pattern of sterna as present in E. (C.) insularis (Fig. 31J).

***Taxonomy*.** This species was described based on male and female subimagines and imagines from the Nakchivan Autonomous Republic (Tshernova 1938). The type series is deposited in IZ (Kluge 1995). The larva was described by Sinitshenkova (1976) based on material collected in Georgia, Russia (the central Greater Caucasus), Armenia and the type locality. Larvae were identified as species *znojkoi*, based on the proximity of its type locality and the similarity of markings on abdominal terga. However, the description of larva is confusing, because the larva of E. (C.) znojkoi was erroneously described under the name E. (C.) caucasicus by Sinitshenkova (1976) (Braasch, 1980). Therefore, the larva described by Sinitshenkova (1976) as E. (C.) znojkoi should belong to a different species. Its diagnostic characters correspond to those of E. (C.) magnus that was later described by Braasch (1978). These characters include: (i) body length: Tshernova (1938) noted 9.5–12 mm for imagines of species E. (C.) znojkoi; contrary to Sinitshenkova (1976) who noted 14–19 mm for the larvae. Larvae of species *magnus* exhibit 20–24 mm as described by Braasch (1978); (ii) shape of head: trapezoidal head with rounded edges as figured by Sinitshenkova (1976) is typical for E. (C.) magnus (Fig. 10D, E), not to E. (C.) znojkoi with more angular edges of head (Fig. 7D); (iii) setation of labrum: the shape of labrum and dense setae on its dorsal surface as figured by Sinitshenkova (1976) is characteristic for E. (C.) magnus (Fig. 11A); (iv) coloration of abdominal sterna: an absence of coloration on abdominal sterna as described by Sinitshenkova (1976) is typical for E. (C.) magnus (Fig. 10J); E. (C.) znojkoi possess reddish sterna and gills.

***Distribution*.**E. (C.) znojkoi is considered as a species complex containing several lineages (Hrivniak et al. 2020b). They are distributed in the Pontic Mts. in Turkey (*Caucasiron* sp. 5 in Hrivniak et al. 2020b), the Alborz Mts. in Iran (*Caucasiron* sp. 4 in Hrivniak et al. 2020b), and the Lesser Caucasus in Georgian Adjara (*Caucasiron* sp. 6 in Hrivniak et al. 2020b). The lineages are not formally described now and fall into the group E. (C.) znojkoi s. l. in this identification guide.

#### Epeorus (Caucasiron) magnus

Taxon classificationAnimaliaEphemeropteraHeptageniidae

(Braasch, 1978)

1C3604D6-72A6-5663-972A-A9B8D8047BFB

Figs 9 , 10 , 11

Iron znojkoi Tshernova, 1938; in Sinitshenkova (1976), partim
Iron magnus Braasch, 1978
Epeorus (Iron) magnus (Braasch, 1978); in Kluge (1988)
Epeorus (Caucasiron) magnus (Braasch, 1978); in Kluge (1997b)

##### Type locality.

Russia, Krasnodar krai, western Caucasus, Sochi River (20 km above Sochi; 800 m a.s.l.).

##### Distribution.

**Figure 9.**
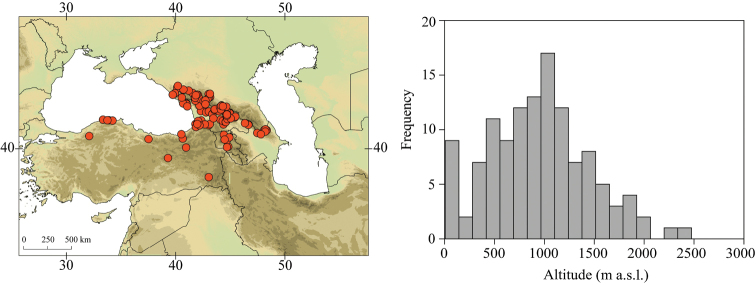
Geographical (left) and vertical (right) distribution of Epeorus (Caucasiron) magnus.

Georgia, south-western Russia, Azerbaijan, Armenia, Turkey (Fig. 9). One of the most widespread species in the Caucasus.

##### Habitat.

Larvae inhabit streams and rivers of various sizes, from larger braided low-altitude rivers to small streams at high altitude. Altitudinal range of sampling sites 6–2474 m a.s.l. (Fig. 9). Most frequently found at low and middle altitude. Often syntopic with E. (C.) znojkoi.

##### Main morphological diagnostics of larvae.

**Figure 10.**
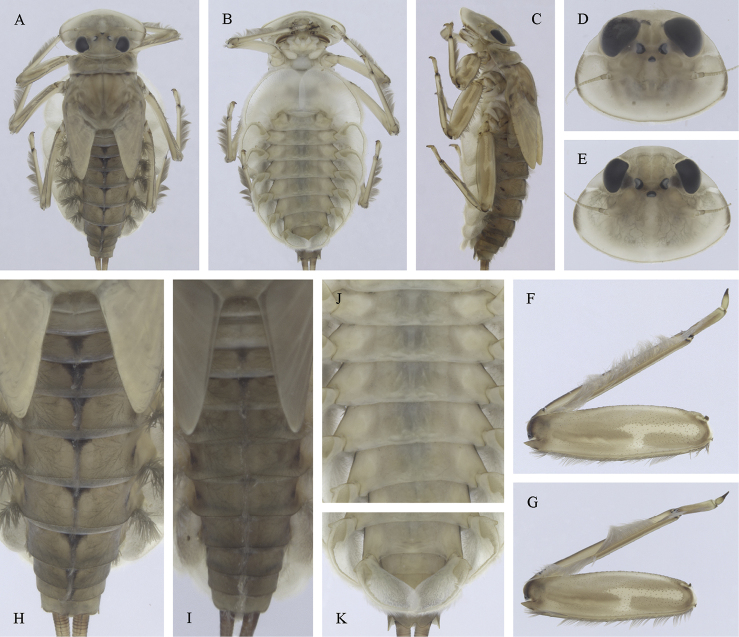
Epeorus (Caucasiron) magnus, larva: **A** habitus in dorsal view **B** habitus in ventral view **C** habitus in lateral view **D** head of male in dorsal view **E** head of female in dorsal view **F, G** middle leg in dorsal view **H, I** abdominal terga **J** abdominal sterna II–VI **K** gills VII (in natural position from ventral view).

**Figure 11.**
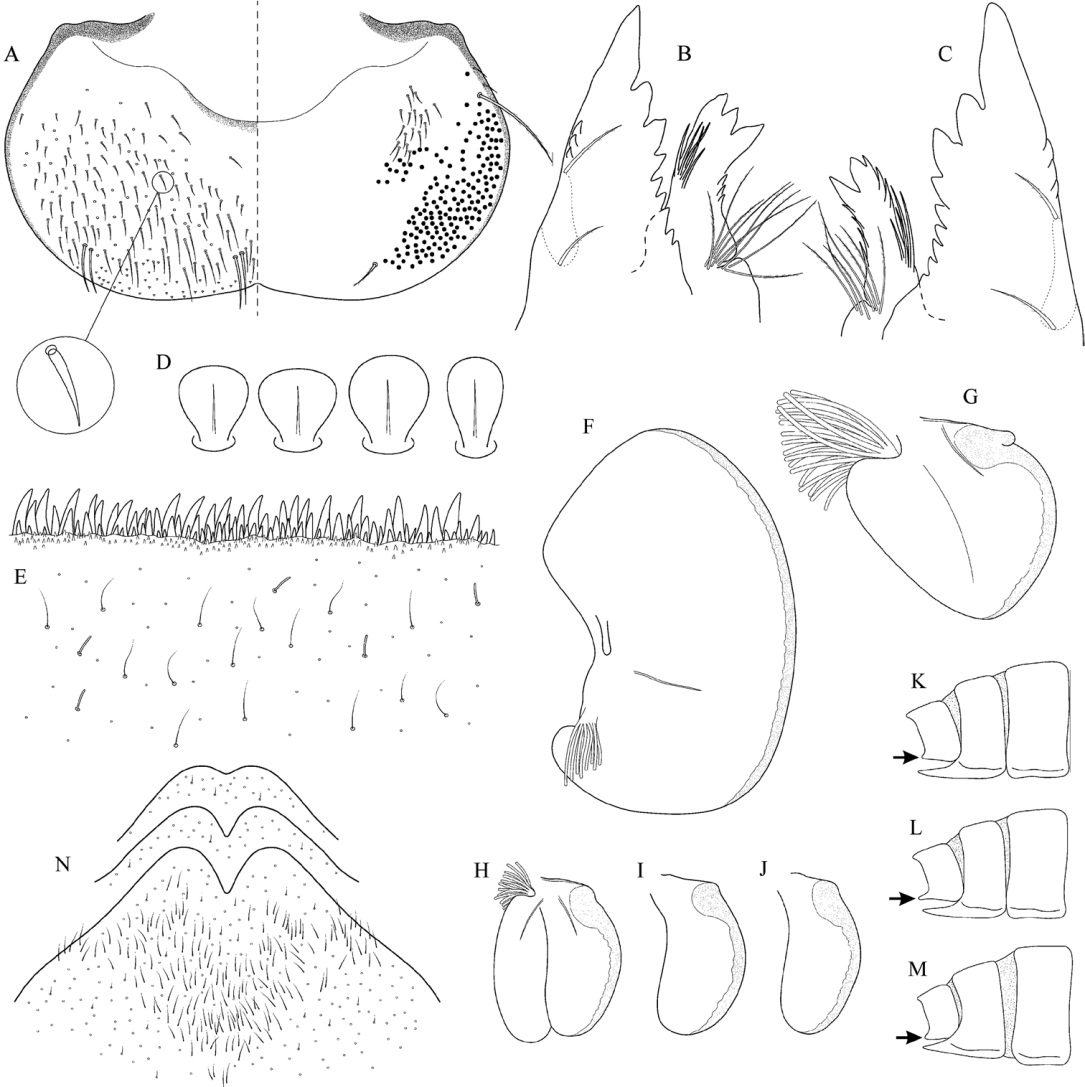
Epeorus (Caucasiron) magnus, larva: **A** labrum (left half in dorsal view right half in ventral view) with detail of bristle-like seta **B** incisors of left mandible **C** incisors of right mandible **D** setae on dorsal surface of femora **E** surface and posterior margin of abdominal tergum VII **F** gill I **G** gill III **H** gill VII (flattened on slide) **I, J** gill VII (in natural position from ventral view) variability in shape **K–M** abdominal segments VIII–X in lateral view (arrow points on postero-lateral projection) **N** sternum IX of female with observed variability.

(i) shape of head in male and female oval, trapezoidal (Fig. 10D, E); (ii) tergum X with well-developed postero-lateral projections (Fig. 11K–M, arrows), sporadically poorly developed; (iii) abdominal sterna without coloration pattern (Fig. 10B, J); (iv) abdominal terga V–VII with triangular medial macula (Fig. 10H), sporadically poorly visible (Fig. 10I); (v) femora without medial hypodermal spot (Fig. 10F, G); (vi) dorsal surface of labrum densely covered by bristle-like setae (Fig. 11A); (v) setae on abdominal terga hair-like (Fig. 11E); (vi) gill plates III with well-developed projection (Fig. 11G); (vii) denticles along posterior margin of tergum VII strongly sclerotized, dense and curved (Fig. 11E).

##### Remarks.

***Morphology*.** The largest species occurring in the Caucasus. The body size of larvae 20–24 mm, cerci 20–22 mm (Braasch 1978).

***Taxonomy*.** Original description based on the larvae from Russia (western Caucasus) (Braasch 1978). The type series is currently deposited in the collection of Stuttgart State Museum of Natural History, Stuttgart, Germany (SMNS). Imagines (male and female) and female subimago described by Braasch (1980) based on material from Russia, Armenia and Georgia. We assume the larva of E. (C.) magnus was erroneously described under the name *znojkoi* by Sinitshenkova (1976) (see remarks to E. (C.) znojkoi s. l. for details).

#### Epeorus (Caucasiron) nigripilosus

Taxon classificationAnimaliaEphemeropteraHeptageniidae

(Sinitshenkova, 1976)

0DAD0969-70C1-5101-B21F-07825305E2C0

Figs 12 , 13 , 14

Iron nigripilosus Sinitshenkova, 1976
Epeorus (Iron) nigripilosus (Sinitshenkova, 1976); in Kluge (1995)
Epeorus (Caucasiron) nigripilosus (Sinitshenkova, 1976); in Kluge (2004)

##### Type locality.

Georgia, Mtskheta-Mtianeti Region, Kistinka (= Khde, Khdistkhali) River (along the Georgian Military Road, 1300 m a.s.l.).

##### Distribution.

**Figure 12.**
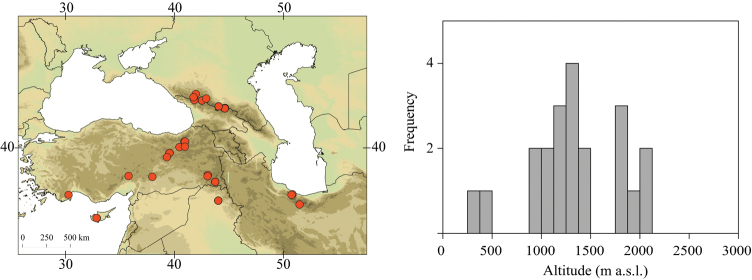
Geographical (left) and vertical (right) distribution of Epeorus (Caucasiron) nigripilosus.

Georgia, south-western Russia, Turkey, Cyprus Island, northern Iraq, northern Iran (Fig. 12).

##### Habitat.

Larvae inhabit small streams and rivers at low to high altitude. Altitudinal range of sampling sites 280–2100 m a.s.l. (Fig. 12). Most frequently found above 1000 m a.s.l.

##### Main morphological diagnostics of larvae.

**Figure 13.**
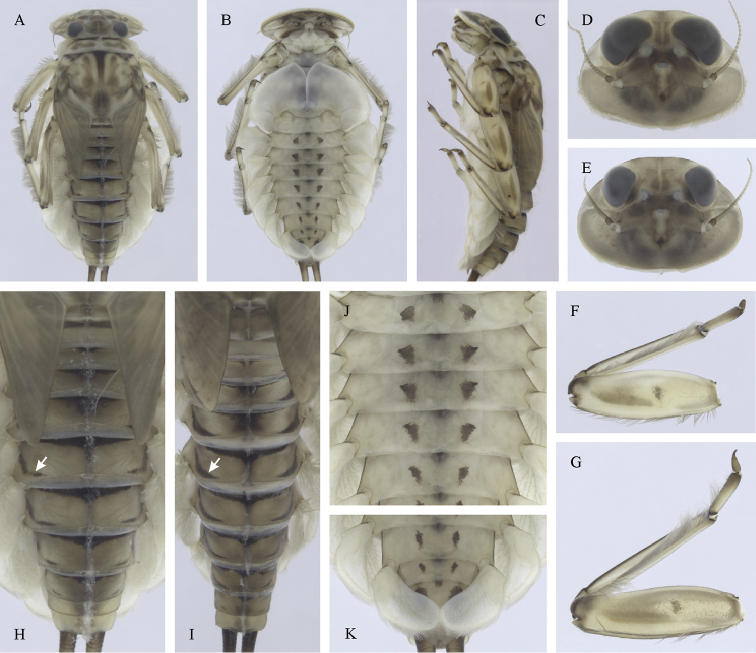
Epeorus (Caucasiron) nigripilosus, larva: **A** habitus in dorsal view **B** habitus in ventral view **C** habitus in lateral view **D** head of male in dorsal view **E** head of female in dorsal view **F, –G** middle leg in dorsal view **H, –I** abdominal terga (arrows point on dorso-posteriorly extended lateral stripes) **J** abdominal sterna II–VI **K** gills VII (in natural position from ventral view).

**Figure 14.**
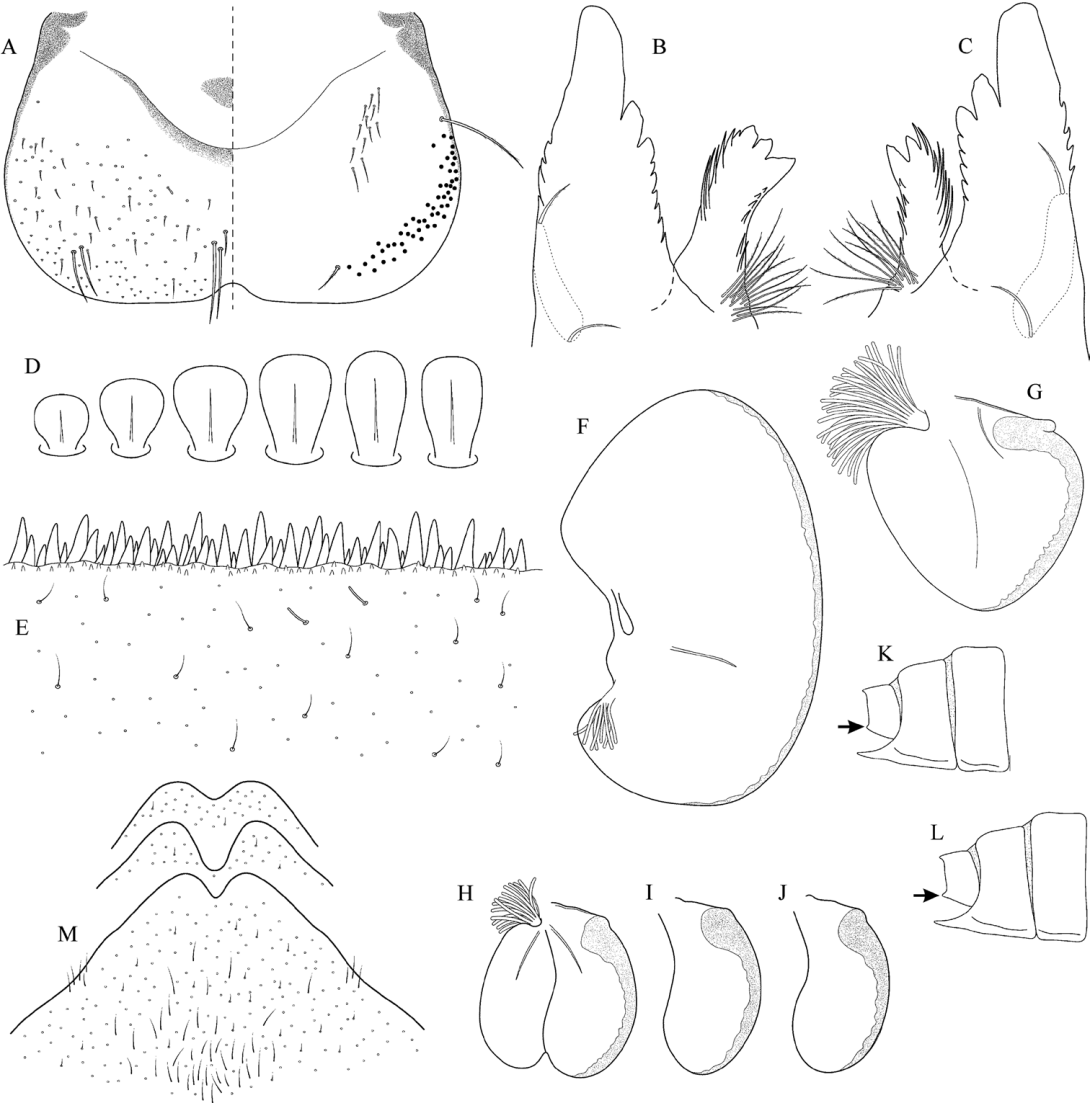
Epeorus (Caucasiron) nigripilosus, larva: **A** labrum (left half in dorsal view right half in ventral view) **B** incisors of left mandible **C** incisors of right mandible **D** setae on dorsal surface of femora **E** surface and posterior margin of abdominal tergum VII **F** gill I **G** gill III **H** gill VII (flattened on slide) **I, J** gill VII (in natural position from ventral view) variability in shape **K, L** abdominal segments VIII–X in lateral view (arrows point on postero-lateral projection) **M**, sternum IX of female with observed variability.

(i) abdominal sterna II–VI with a pair of triangular spots; nerve ganglia often with spots (Fig. 13B, J); (ii) abdominal terga V–VII with lateral stripes extended dorso-posteriorly (Fig. 13H, I, arrows); (iii) tergum X with postero-lateral projections (Fig. 14K, L, arrows); (iv) femora with rounded medial hypodermal spot (Fig. 13F, G); (v) setae on abdominal terga hair-like (Fig. 14E); (vi) denticles along posterior margin of tergum VII strongly sclerotized and dense (Fig. 14E); (vii) gill plates VII (in natural position from ventral view) wide (Figs 13K, 14H–J); (viii) gill plates III with developed projection (Fig. 14G).

##### Remarks.

***Taxonomy*.** This species was described based on larvae from Georgia (Kistinka River) (Sinitshenkova 1976). Type series is deposited in IZ (Kluge 1995). Male imago was described by Braasch (1979) based on the material from the western Caucasus (Teberda River) associated with larvae according to similar coloration of abdominal terga and sterna. Female imago not described. Male genitalia similar to E. (C.) caucasicus according to Braasch (1979).

#### Epeorus (Caucasiron) alpestris

Taxon classificationAnimaliaEphemeropteraHeptageniidae

(Braasch, 1979)

D1F98C55-5DDD-5F8D-AA93-96C1A71D4118

Figs 15 , 16 , 17

Iron alpestris Braasch, 1979
Epeorus (Iron) alpestris (Braasch, 1979); in Kluge (1988)
Epeorus (Caucasiron) alpestris (Braasch, 1979); in Kluge (1997b)

##### Type locality.

Russia, The Karachay-Cherkess Republic, western Greater Caucasus, Teberda (Glacier Alibek – stream, 1800–1900 m a.s.l.).

##### Distribution.

**Figure 15.**
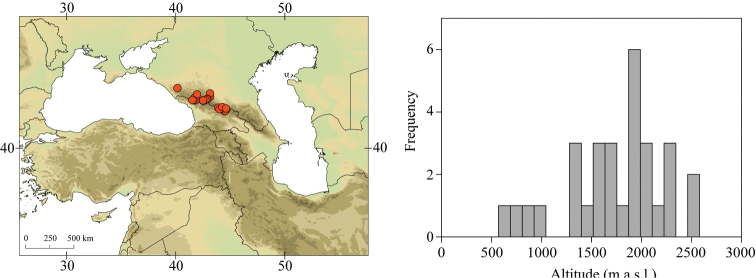
Geographical (left) and vertical (right) distribution of Epeorus (Caucasiron) alpestris.

Georgia, south-western Russia. Species endemic to the Greater Caucasus (Fig. 15).

##### Habitat.

Larvae inhabit small streams and rivers at middle and high altitude in the western and central Greater Caucasus. Altitudinal range of sampling sites 570–2580 m a.s.l (Fig. 15). Most frequently found at altitudes above 1200 m a.s.l. Often syntopic with E. (C.) soldani and at higher altitude with E. (C.) sinitshenkovae.

##### Main morphological diagnostics of larvae.

**Figure 16.**
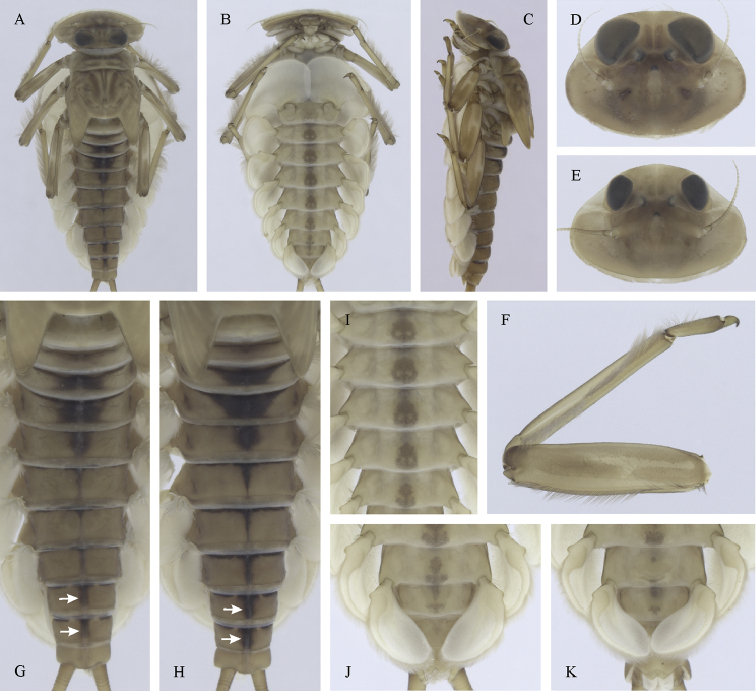
Epeorus (Caucasiron) alpestris, larva: **A** habitus in dorsal view **B** habitus in ventral view **C** habitus in lateral view **D** head of male in dorsal view **E** head of female in dorsal view **F** middle leg in dorsal view **G, H** abdominal terga (arrows point on widened medial maculae) **I** abdominal sterna II–VI **J, K** gills VII (in natural position from ventral view).

**Figure 17.**
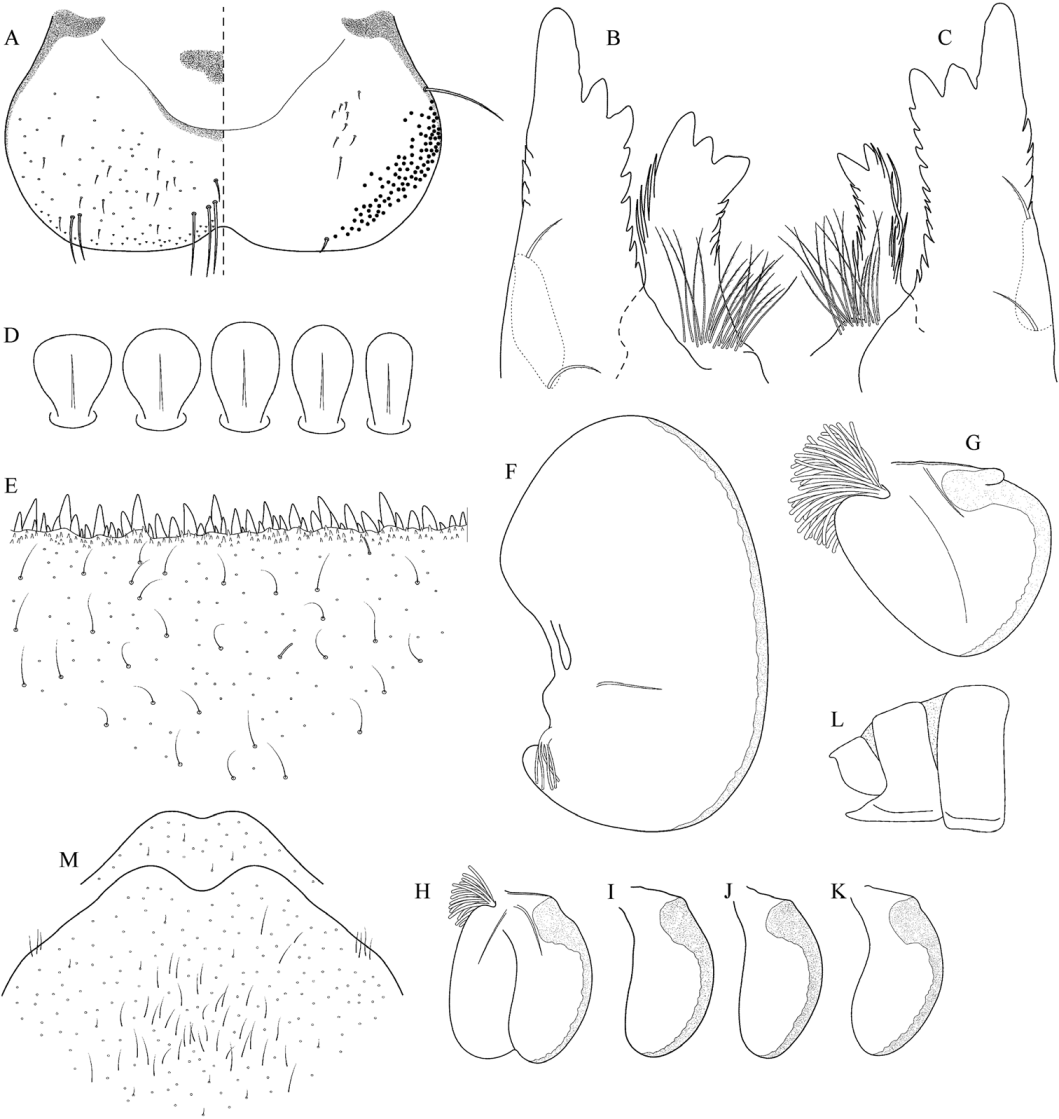
Epeorus (Caucasiron) alpestris, larva: **A** labrum (left half in dorsal view right half in ventral view) **B** incisors of left mandible **C** incisors of right mandible **D** setae on dorsal surface of femora **E** surface and posterior margin of abdominal tergum VII **F** gill I **G** gill III **H** gill VII (flattened on slide) **I–K** gill VII (in natural position from ventral view) variability in shape **L** abdominal segments VIII–X in lateral view **M** sternum IX of female with observed variability.

(i) abdominal terga V–VII with narrow stripe-like medial macula; widened on terga VIII–IX (Fig. 16G, H, arrows); (ii) abdominal sterna II–VI with rounded medial macula (Fig. 16B, I); (iii) femora without medial hypodermal spot (Fig. 16F); (iv) tergum X without postero-lateral projections (Fig. 17L); (v) gill plates III with well-developed projection (Fig. 17G); (vi) setae on abdominal terga hair-like (Fig. 17E); (vii) dorsal surface of labrum with sparse hair-like setae (Fig. 17A); (viii) gill plates VII (in natural position of ventral view) wide (Figs 16J, K, 17H–K).

##### Remarks.

***Taxonomy*.** This species was described based on the male imago and larva from western Greater Caucasus (Braasch 1979). The type series is currently deposited in SMNS. Imagines and larvae were associated based on the coloration of abdomen. Female imago not described.

#### Epeorus (Caucasiron) soldani

Taxon classificationAnimaliaEphemeropteraHeptageniidae

(Braasch, 1979)

CEB8A4B6-F04F-5575-AC0A-AE35F095C786

Figs 18 , 19 , 20

Iron soldani Braasch, 1979
Epeorus (Iron) soldani (Braasch, 1979); in Kluge (1988)
Epeorus (Caucasiron) soldani (Braasch, 1979); in Kluge (1997b)

##### Type locality.

Russia, The Karachay-Cherkess Republic, western Greater Caucasus, Teberda (Glacier Alibek – stream, 1800–1900 m a.s.l.).

##### Distribution.

**Figure 18.**
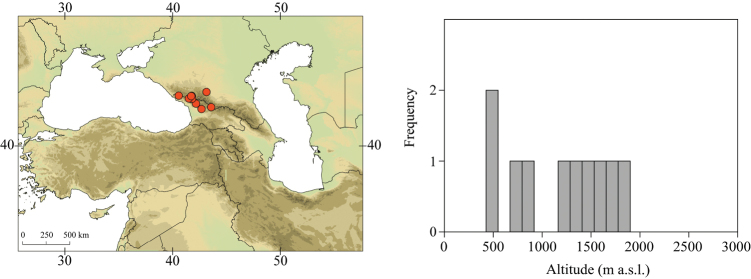
Geographical (left) and vertical (right) distribution of Epeorus (Caucasiron) soldani.

Georgia, south-western Russia. Species endemic to the Greater Caucasus (Fig. 18).

##### Habitat.

Larvae inhabit small streams and rivers at middle and high altitudes in the western and central Greater Caucasus. Frequently found above 1000 m a.s.l. Altitudinal range of sampling sites 426–1900 m a.s.l. (Fig. 18). Often syntopic with E. (C.) alpestris and E. (C.) sinitshenkovae.

##### Main morphological diagnostics of larvae.

**Figure 19.**
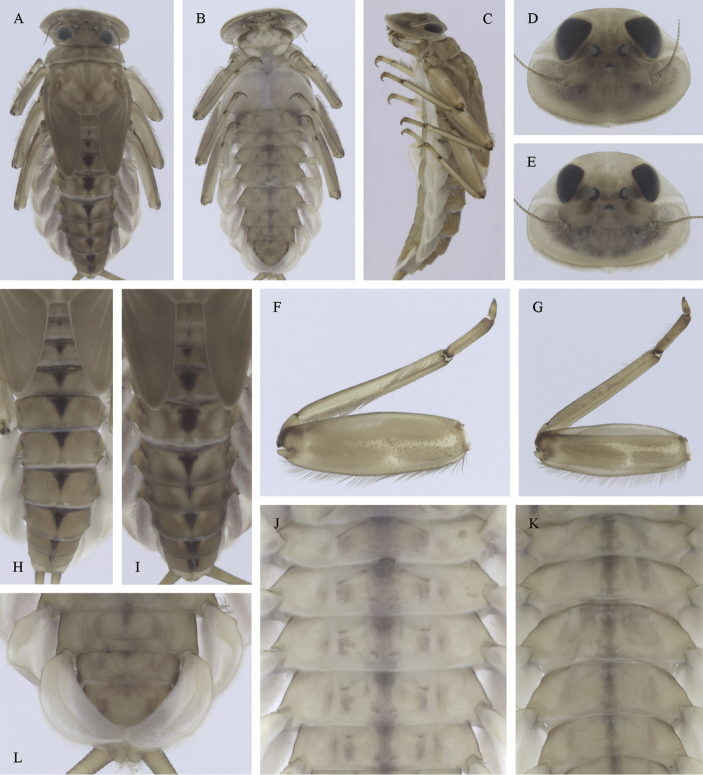
Epeorus (Caucasiron) soldani, larva: **A** habitus in dorsal view **B** habitus in ventral view **C** habitus in lateral view **D** head of male in dorsal view **E** head of female in dorsal view **F, G** middle leg in dorsal view **H, I** abdominal terga **J, K** abdominal sterna II–VI **L** gills VII (in natural position from ventral view).

**Figure 20.**
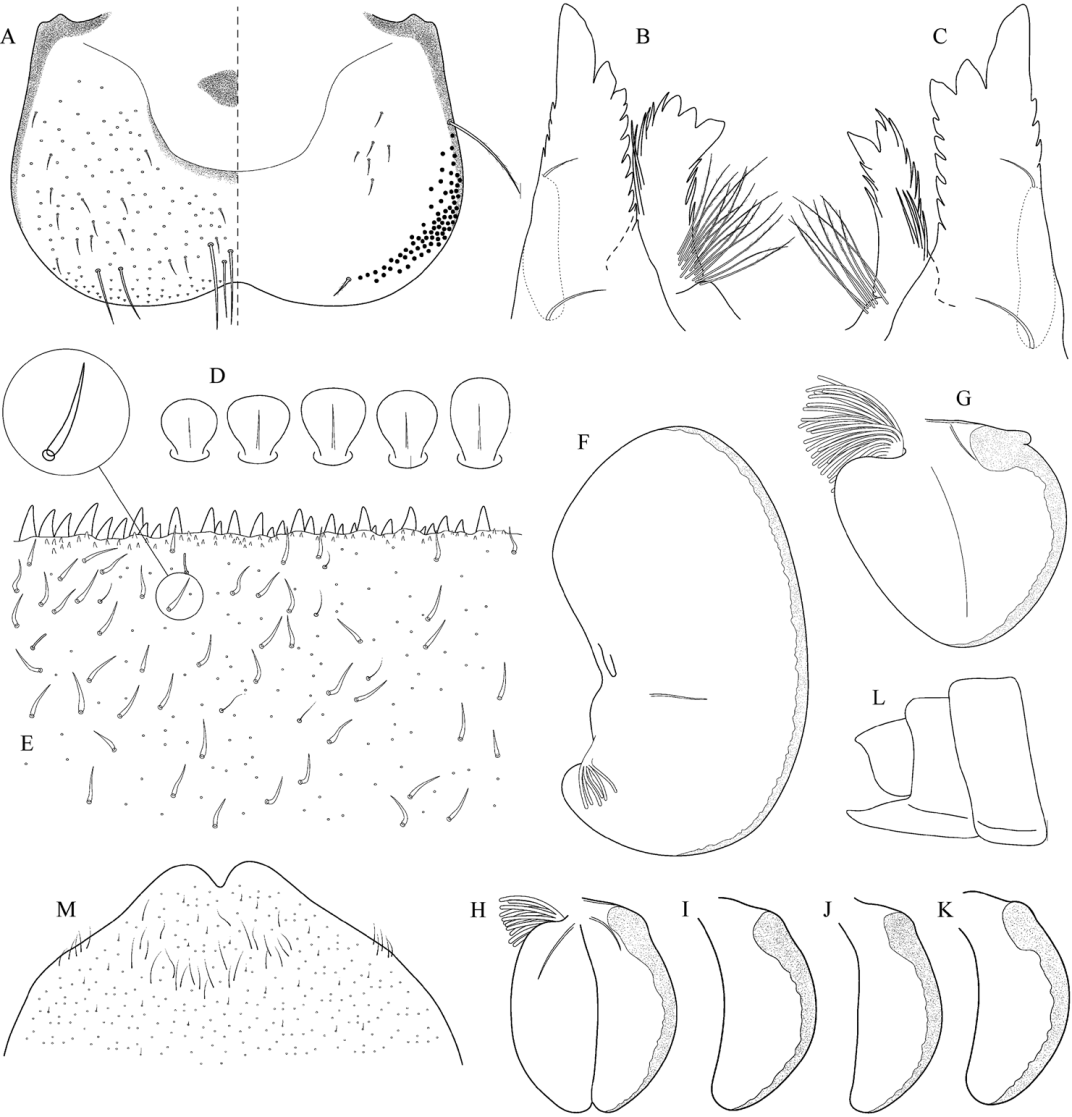
Epeorus (Caucasiron) soldani, larva: **A** labrum (left half in dorsal view right half in ventral view) **B** incisors of left mandible **C** incisors of right mandible **D** setae on dorsal surface of femora **E** surface and posterior margin of abdominal tergum VII with detail of basally wide seta **F** gill I **G** gill III **H** gill VII (flattened on slide) **I–K** gill VII (in natural position from ventral view) variability in shape **L** abdominal segments VIII–X in lateral view **M** sternum IX of female.

(i) abdominal terga V–VII with well-defined triangular medial maculae (Fig. 19H, I); (ii) abdominal sterna II–VI either without pattern or with indistinct pattern as on Fig. 19J, K; (iii) setae on abdominal terga wide at base (Fig. 20E); (iv) femora without medial hypodermal spot (Fig. 19F, G); (v) tergum X without postero-lateral projections (Fig. 20L); (vi) gill plates III with well-developed projection (Fig. 20G); (vii) gill plates VII (in natural position of ventral view) narrow (Figs 19L; 20H–K); (viii) denticles along posterior margin of tergum VII relatively sparse and triangular (Fig. 20E).

##### Remarks.

***Taxonomy*.** This species was described based on male imago and larva from the western Greater Caucasus (Braasch 1979). The type series is currently deposited in SMNS. Larva associated with imago based on the coloration of abdomen. Female imago not described. The lineage *Caucasiron* sp. 7 detected by Hrivniak et al. (2020b) is distributed in Georgia and morphologically corresponds to E. (C.) soldani. Therefore, E. (C.) soldani may represent a species complex.

#### Epeorus (Caucasiron) iranicus

Taxon classificationAnimaliaEphemeropteraHeptageniidae

(Braasch & Soldán, 1979)

6305A3AA-5CF0-5A2C-AED4-682F2331F5B4

Figs 21 , 22 , 23

Iron caucasicus iranicus Braasch & Soldán, 1979
Epeorus (Caucasiron) caucasicus iranicus (Braasch & Soldán, 1979); in Bojková et al. (2018)
Epeorus (Caucasiron) iranicus (Braasch & Soldán, 1979); in Hrivniak et al. (2020b)

##### Type locality.

Iran, Tehran Province, river in the Darban-Tal (Darban Valley), 2100 m a.s.l.

##### Distribution.

**Figure 21.**
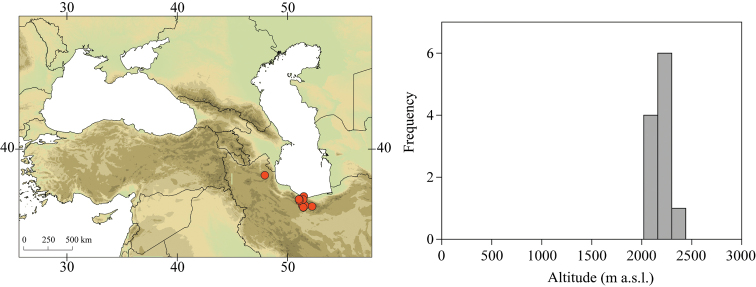
Geographical (left) and vertical (right) distribution of Epeorus (Caucasiron) iranicus.

Northern Iran. Species endemic to the Alborz Mountains (Fig. 21).

##### Habitat.

Larvae inhabit streams at altitudes above 2000 m a.s.l. in the western and central Alborz. Altitudinal range of sampling sites 2020–2440 m a.s.l. (Fig. 21). Often syntopic with E. (C.) alborzicus.

##### Main morphological diagnostics of larvae.

**Figure 22.**
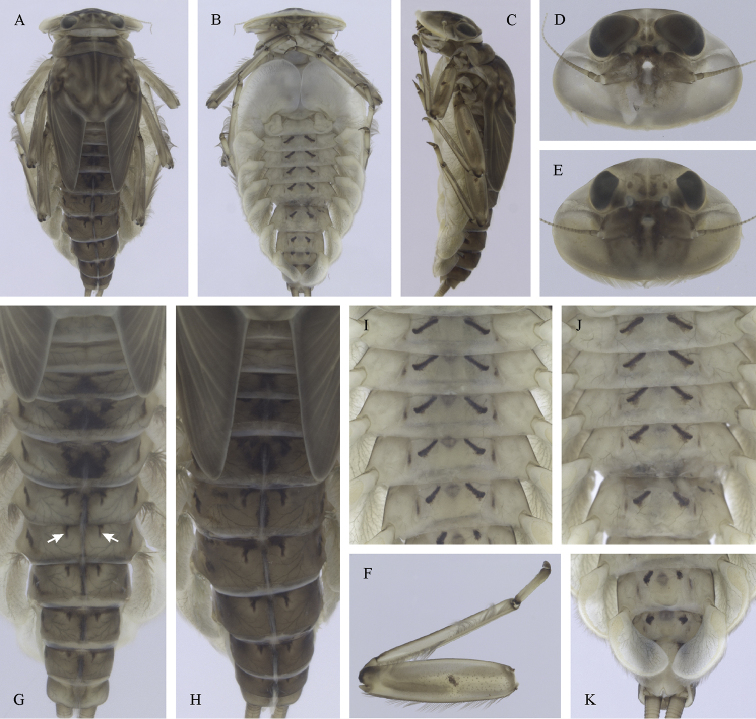
Epeorus (Caucasiron) iranicus, larva: **A** habitus in dorsal view **B** habitus in ventral view **C** habitus in lateral view **D** head of male in dorsal view **E** head of female in dorsal view **F** middle leg in dorsal view **G, H** abdominal terga (arrows point antero-lateral stripes of medial macula) **I, J** abdominal sterna II–VI **K** gills VII (in natural position from ventral view).

**Figure 23.**
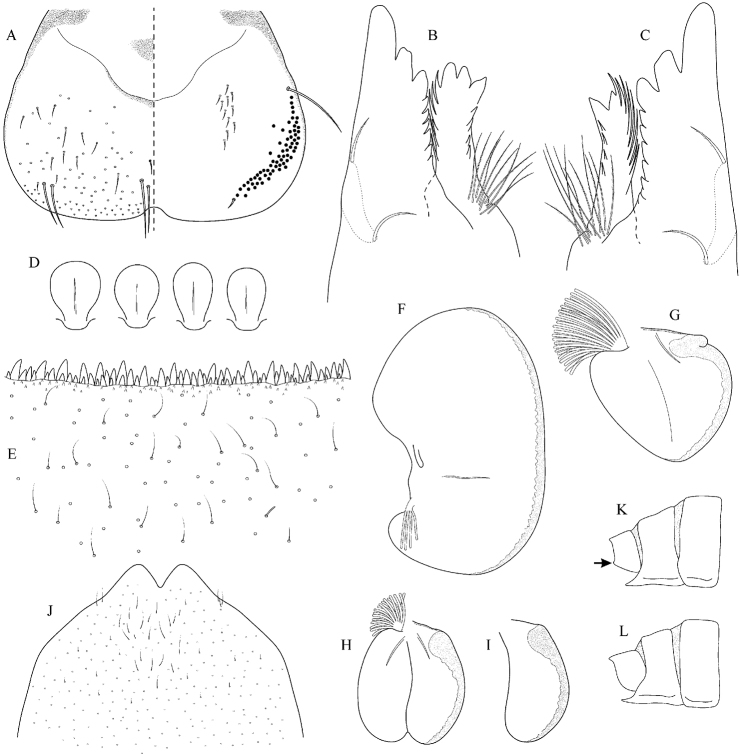
Epeorus (Caucasiron) iranicus, larva: **A** labrum (left half in dorsal view right half in ventral view) **B** incisors of left mandible **C** incisors of right mandible **D** setae on dorsal surface of femora **E** surface and posterior margin of abdominal tergum VII **F** gill I **G** gill III **H** gill VII (flattened on slide) **I** gill VII (in natural position from ventral view) **J** sternum IX of female **K, L** abdominal segments VIII–X in lateral view (arrow points on postero-lateral projection).

(i) abdominal sterna II–VI with a pair of oblique stripes; nerve ganglia often with stripes or spots (Fig. 22B, I, J); (ii) abdominal terga V–VII with stripe-like medial macula and a pair of distinct antero-lateral stripes (Fig. 22G, arrows); (iii) femora with rounded medial hypodermal spot (Fig. 22F); (iv) gill III with well-developed projection (Fig. 23G); (v) setae on abdominal terga hair-like (Fig. 23E); (vi) tergum X with poorly developed postero-lateral projections (Fig. 23K, arrow) or without postero-lateral projections (Fig. 23L).

##### Remarks.

***Morphology*.** Coloration pattern on abdominal sterna as in E. (C.) caucasicus (Fig. 4J), similar pattern in E. (C.) zagrosicus (Fig. 46I).

***Taxonomy*.** This species was described as a subspecies of E. (C.) caucasicus based on larvae collected in the Alborz Mts. (Braasch and Soldán 1979). Elevated to species level by Hrivniak et al. (2020b) based on a phylogenetic analysis of all Caucasian Epeorus (Caucasiron) species. The holotype probably lost. Paratypes are currently deposited in SMNS and Biology Centre of the Czech Academy of Sciences, Institute of Entomology, České Budějovice, Czech Republic (IECA). Imagines and subimagines not described.

#### Epeorus (Caucasiron) sinitshenkovae

Taxon classificationAnimaliaEphemeropteraHeptageniidae

(Braasch & Zimmerman, 1979)

1D217FF0-331E-5DFE-A23A-260AD89262AD

Figs 24 , 25 , 26

Iron sinitshenkovae Braasch & Zimmermann, 1979
Epeorus (Iron) sinitshenkovae (Braasch & Zimmermann, 1979); in Kluge (1995)
Epeorus (Caucasiron) sinitshenkovae (Braasch & Zimmermann, 1979); in Kluge (2004)

##### Type locality.

Russia, the Kabardino-Balkarian Republic, central Greater Caucasus, right tributary of Dongoserun (Donguz-Orun-Baksan) River (2100 m a.s.l.).

##### Distribution.

**Figure 24.**
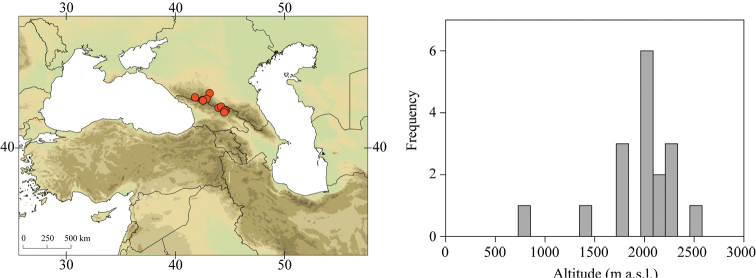
Geographical (left) and vertical (right) distribution of Epeorus (Caucasiron) sinitshenkovae.

Georgia, south-western Russia. Species endemic to the Greater Caucasus (Fig. 24).

##### Habitat.

Larvae inhabit small streams and rivers at middle and high altitude in the western and central Greater Caucasus. Altitudinal range of sampling sites 760–2580 m a.s.l. (Fig. 24). Most frequently found above 1800 m a.s.l. Often syntopic with E. (C.) alpestris and at lower altitude with E. (C.) soldani.

##### Main morphological diagnostics of larvae.

**Figure 25.**
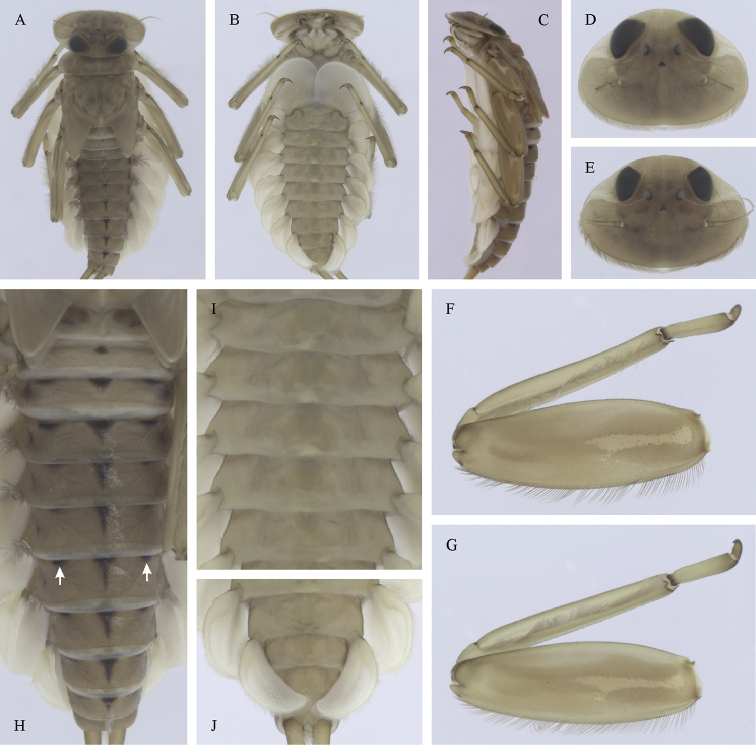
Epeorus (Caucasiron) sinitshenkovae, larva: **A** habitus in dorsal view **B** habitus in ventral view **C** habitus in lateral view **D** head of male in dorsal view **E** head of female in dorsal view **F, G** middle leg in dorsal view H abdominal terga (arrows point on anterolateral spots) **I** abdominal sterna II–VI **J** gills VII (in natural position from ventral view).

**Figure 26.**
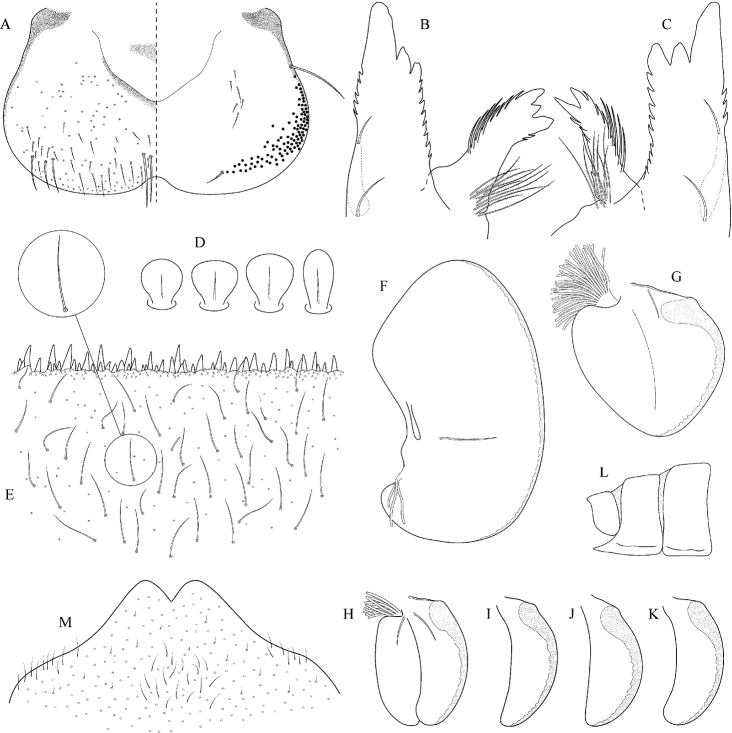
Epeorus (Caucasiron) sinitshenkovae, larva: **A** labrum (left half in dorsal view right half in ventral view) **B** incisors of left mandible **C** incisors of right mandible **D** setae on dorsal surface of femora **E** surface and posterior margin of abdominal tergum VII with detail of slightly widened elongated seta **F** gill I **G** gill III **H** gill VII (flattened on slide) **I–K** gill VII (in natural position from ventral view) variability in shape **L** abdominal segments VIII–X in lateral view **M** sternum IX of female.

(i) abdominal terga V–VII with narrowed triangular medial macula and a pair of anterolateral spots (Fig. 25H; arrows); (ii) abdominal sterna without coloration pattern (Fig. 25B, I); (iii) femora without medial hypodermal spot (Fig. 25F, G); (iv) gill plates VII (in natural position from ventral view) narrow (Figs 25J, 26H–K); (v) gill plates III with poorly developed projection (Fig. 26G); (vi) setae on abdominal terga not distinctly wide at base, often elongated (Fig. 26E); (vii) tergum X without postero-lateral projections (Fig. 26L).

##### Remarks.

***Taxonomy*.** Original description based on male imago and larva from the Greater Caucasus (Braasch and Zimmermann 1979). The type series is currently deposited in SMNS. Female imago not described in detail. The association of imagines and larvae based on the colour pattern of abdominal terga and sterna in material from the same locality.

#### Epeorus (Caucasiron) longimaculatus

Taxon classificationAnimaliaEphemeropteraHeptageniidae

(Braasch, 1980)

205120CA-5CFD-527D-A25C-585CC257F189

Figs 27 , 28 , 29

Iron longimaculatus Braasch, 1980
Epeorus (Caucasiron) longimaculatus (Braasch, 1980); in Kluge (2004)

##### Type locality.

Georgia, Mtskheta-Mtianeti Region, central Greater Caucasus, tributary of Aragvi River, 3 km above Pasanauri (1400–1500 m a.s.l.).

##### Distribution.

**Figure 27.**
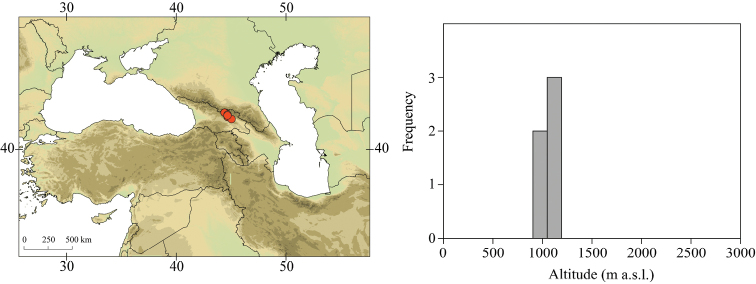
Geographical (left) and vertical (right) distribution of Epeorus (Caucasiron) longimaculatus.

Georgia. Species endemic to the Greater Caucasus (Fig. 27).

##### Habitat.

Larvae inhabit small streams and rivers at middle altitude in the central Greater Caucasus. Altitudinal range of sampling sites 903–1193 m a.s.l. (Fig. 27).

##### Main morphological diagnostics of larvae.

**Figure 28.**
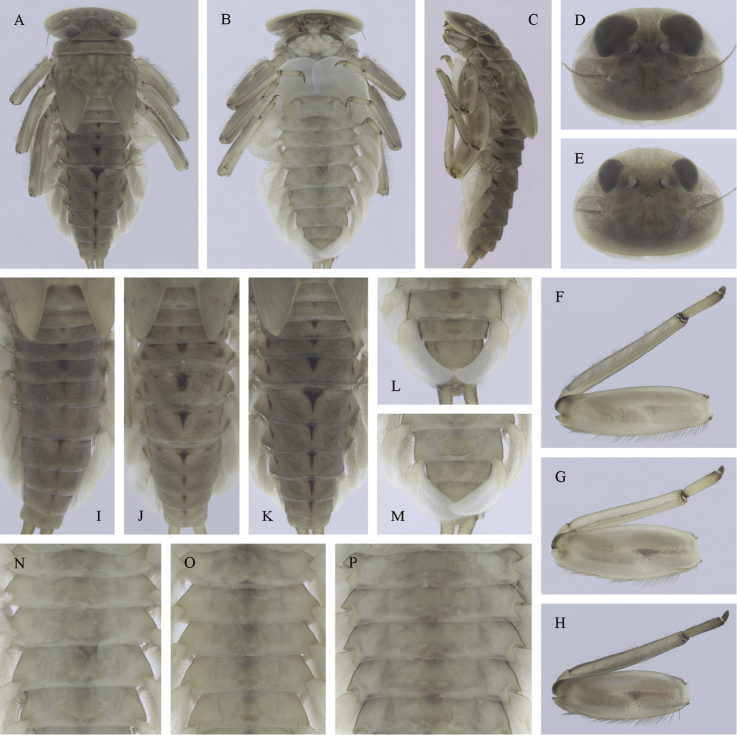
Epeorus (Caucasiron) longimaculatus, larva: **A** habitus in dorsal view **B** habitus in ventral view **C** habitus in lateral view **D** head of male in dorsal view **E** head of female in dorsal view **F–H** middle leg in dorsal view **I–K** abdominal terga **L, M** gills VII (in natural position from ventral view) **N–P** abdominal sterna II–VI.

**Figure 29.**
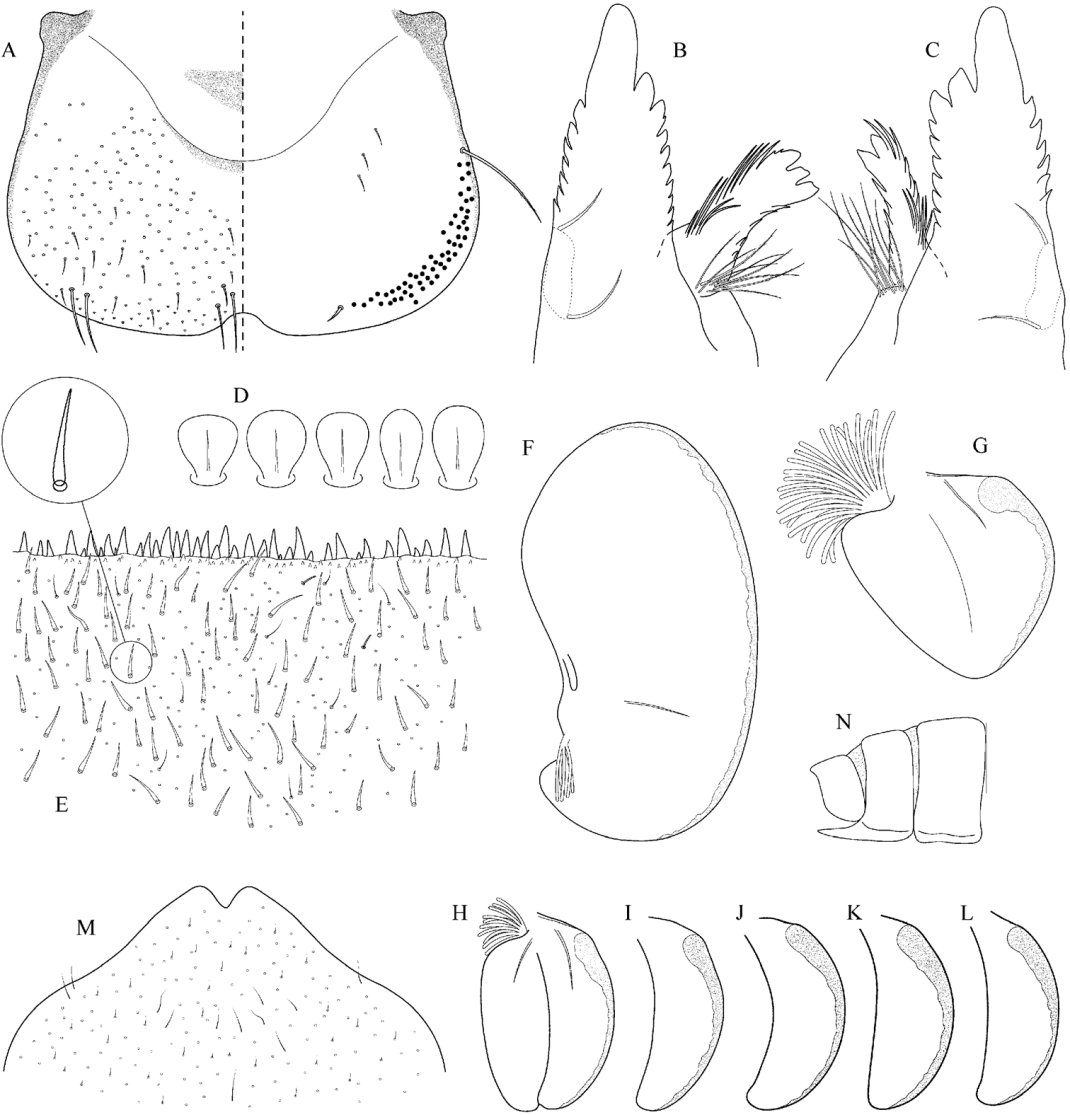
Epeorus (Caucasiron) longimaculatus, larva: **A** labrum (left half in dorsal view right half in ventral view) **B** incisors of left mandible **C** incisors of right mandible **D** setae on dorsal surface of femora **E** surface and posterior margin of abdominal tergum VII with detail of basally wide seta **F** gill I **G** gill III **H** gill VII (flattened on slide) **I–L** gill VII (in natural position from ventral view) variability in shape **M** sternum IX of female **N** abdominal segments VIII–X in lateral view.

(i) femora with elongated medial hypodermal spot (Fig. 28F–H); (ii) setae on abdominal terga wide at base (Fig. 29E); (iii) gill plates III without distinct projection (Fig. 29G); (iv) gill plates VII (in natural position from ventral view) narrow (Figs 28L, M, 29H–L); (v) denticles along posterior margin of tergum VII narrowed (Fig. 29E); (vi) abdominal terga V–VII with narrowed triangular medial macula (Fig. 28I–K); (vii) abdominal sterna without coloration pattern (Fig. 28B, N–P); (viii) tergum X without postero-lateral projections (Fig. 29N); (ix) shape of head of male ellipsoid (Fig. 28D).

##### Remarks.

***Taxonomy*.** This species described based on male subimago and larva collected in central Greater Caucasus (Braasch 1980). The type series is currently deposited in SMNS. Larva associated with the subimago according to the coloration of abdomen. Male and female imagines not described.

#### Epeorus (Caucasiron) insularis

Taxon classificationAnimaliaEphemeropteraHeptageniidae

(Braasch, 1983)

9E76892A-7741-55B1-99D7-D3A5794CE632

Figs 30 , 31 , 32

Iron znojkoi insularis Braasch, 1983
Epeorus (Caucasiron) insularis (Braasch, 1983); in Hrivniak et al. (2020b)

##### Type locality.

Greece, Samos Island, stream east of Pirgos, 37°3'N/26°49'E; 300 m a.s.l.

##### Distribution.

**Figure 30.**
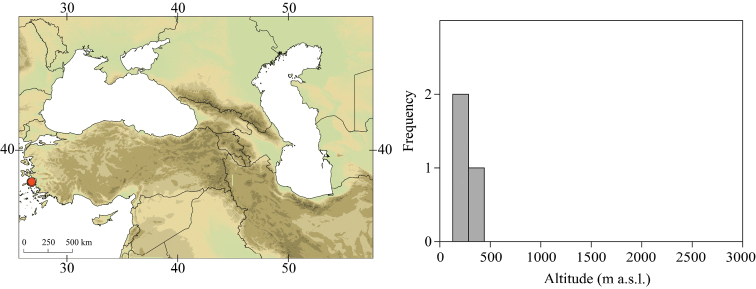
Geographical (left) and vertical (right) distribution of Epeorus (Caucasiron) insularis.

Known only from few sites in Samos Island (Fig. 30).

##### Habitat.

Larvae inhabit small forested streams at 128–440 m a.s.l. (Fig. 30).

##### Main morphological diagnostics of larvae.

**Figure 31.**
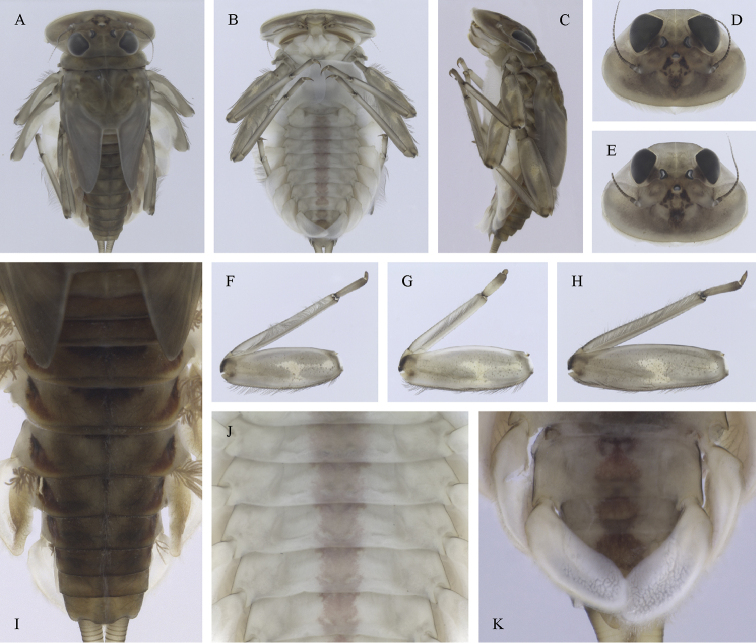
Epeorus (Caucasiron) insularis, larva: **A** habitus in dorsal view **B** habitus in ventral view **C** habitus in lateral view **D** head of male in dorsal view **E** head of female in dorsal view **F–H** middle leg in dorsal view **I** abdominal terga **J** abdominal sterna II–VI **K** gills VII (in natural position from ventral view).

**Figure 32.**
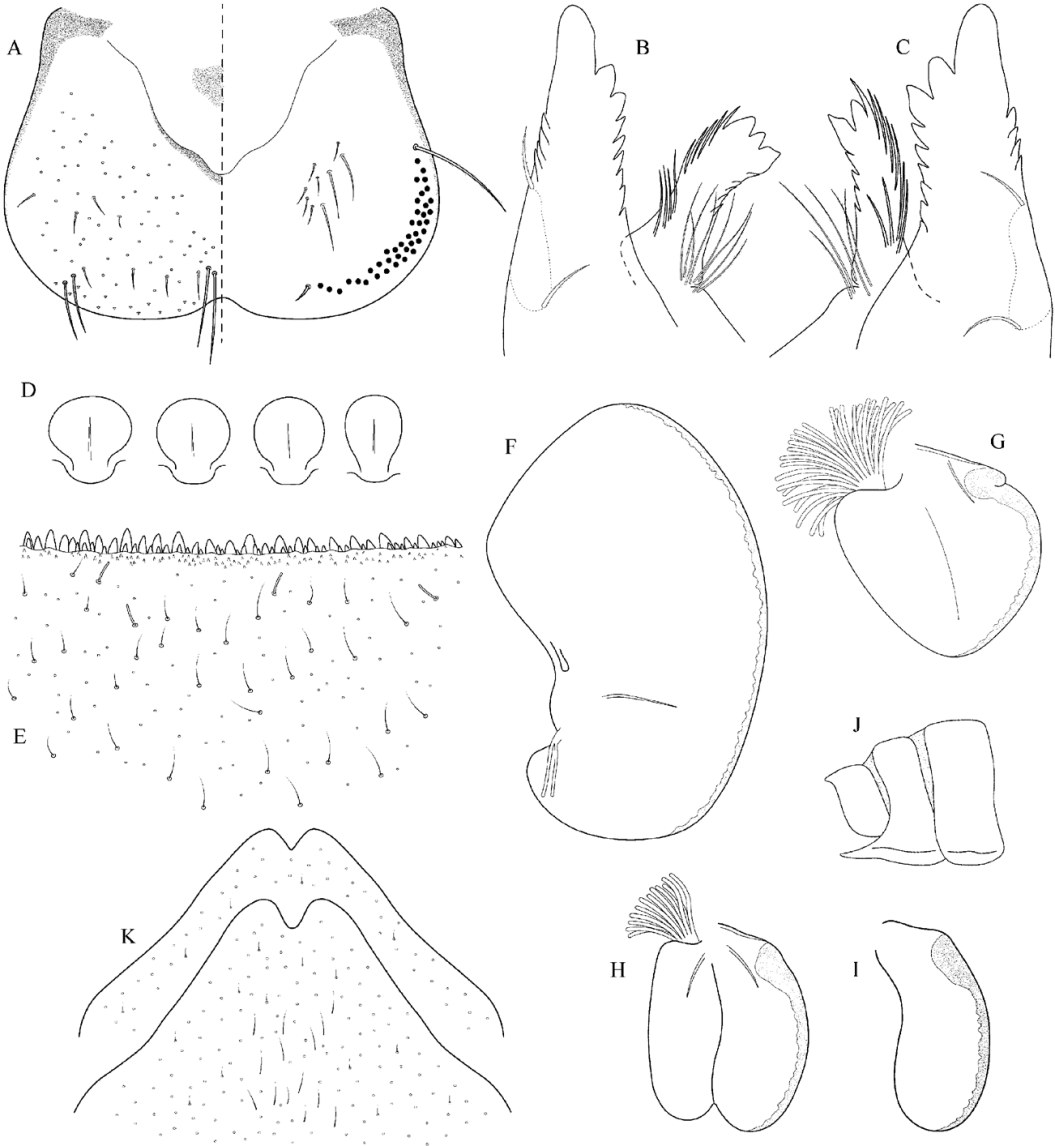
Epeorus (Caucasiron) insularis, larva: **A** labrum (left half in dorsal view right half in ventral view) **B** incisors of left mandible **C** incisors of right mandible **D** setae on dorsal surface of femora **E** surface and posterior margin of abdominal tergum VII **F** gill I **G** gill III **H** gill VII (flattened on slide) **I** gill VII (in natural position from ventral view) **J** abdominal segments VIII–X in lateral view **K** sternum IX of female with observed variability.

(i) abdominal terga V–VII with T-shaped medial macula (Fig. 31I); (ii) abdominal sterna V–VII with reddish to brownish longitudinal stripe (Fig. 31B, J); (iii) tergum X without postero-lateral projections (Fig. 32J); (iv) gill plates VII (in natural position from ventral view) narrow (Figs 31K, 32H, I); (v) gill plates III with well-developed projection (Fig. 32G); (vi) setae on abdominal terga hair-like (Fig. 32E); (vii) denticles along posterior margin of tergum VII relatively short and poorly sclerotized (Fig. 32E).

##### Remarks.

***Morphology*.** Coloration of abdominal terga and sterna as in E. (C.) znojkoi s.l. (Fig. 7N–P).

***Taxonomy*.** This species was described by Braasch (1983) based on imagines as a subspecies of E. (C.) znojkoi. Elevated to species level in Hrivniak et al. (2020b) based on a phylogenetic analysis of all Caucasian Epeorus (Caucasiron) species. The type series is currently deposited in SMNS.

#### Epeorus (Caucasiron) bicolliculatus

Taxon classificationAnimaliaEphemeropteraHeptageniidae

Hrivniak, 2017

1BD0BF09-6C98-5DB7-A8F2-487282BD5A0B

Figs 33 , 34 , 35

Epeorus alpicola (Eaton, 1871); in Türkmen and Kazancı (2015), partim
Epeorus sylvicola (Pictet, 1865); in Türkmen and Kazancı (2015), partim
Epeorus (Caucasiron) sp.; in Martynov et al. (2016)

##### Type locality.

Georgia, Autonomous Republic of Adjara, vicinity of Chakhati village, Kintrishi River; 41°45'43"N/41°58'34"E; 325 m a.s.l.

##### Distribution.

**Figure 33.**
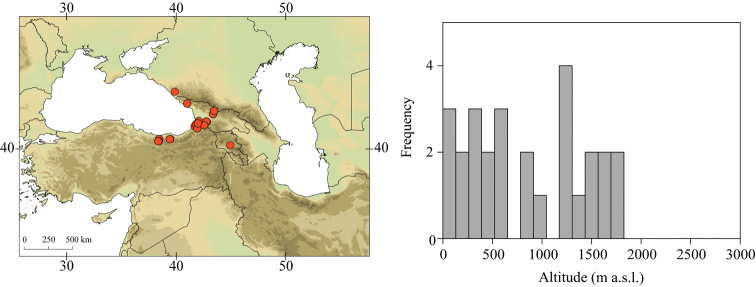
Geographical (left) and vertical (right) distribution of Epeorus (Caucasiron) bicolliculatus.

Georgia, north-eastern Turkey, Armenia, south-western Russia (Fig. 33).

##### Habitat.

Larvae inhabit streams and rivers of different sizes, from to middle-sized rivers at low altitude to small streams at high altitudes. Altitudinal range of sampling sites 40–1804 m a.s.l. (Fig. 33).

##### Main morphological diagnostics of larvae.

**Figure 34.**
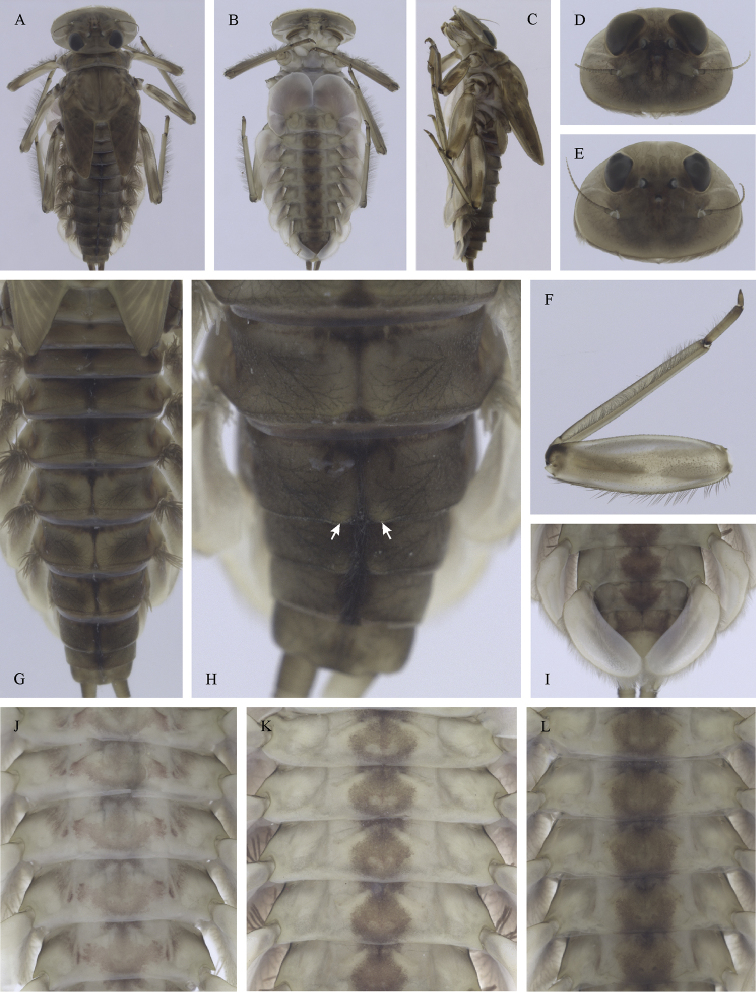
Epeorus (Caucasiron) bicolliculatus, larva: **A** habitus in dorsal view **B** habitus in ventral view **C** habitus in lateral view **D** head of male in dorsal view **E** head of female in dorsal view **F** middle leg in dorsal view **G** abdominal terga **H** abdominal terga VI–X (arrows point on postero-medial protuberances) **I** gills VII (in natural position from ventral view) **J–L** abdominal sterna II–VI.

**Figure 35.**
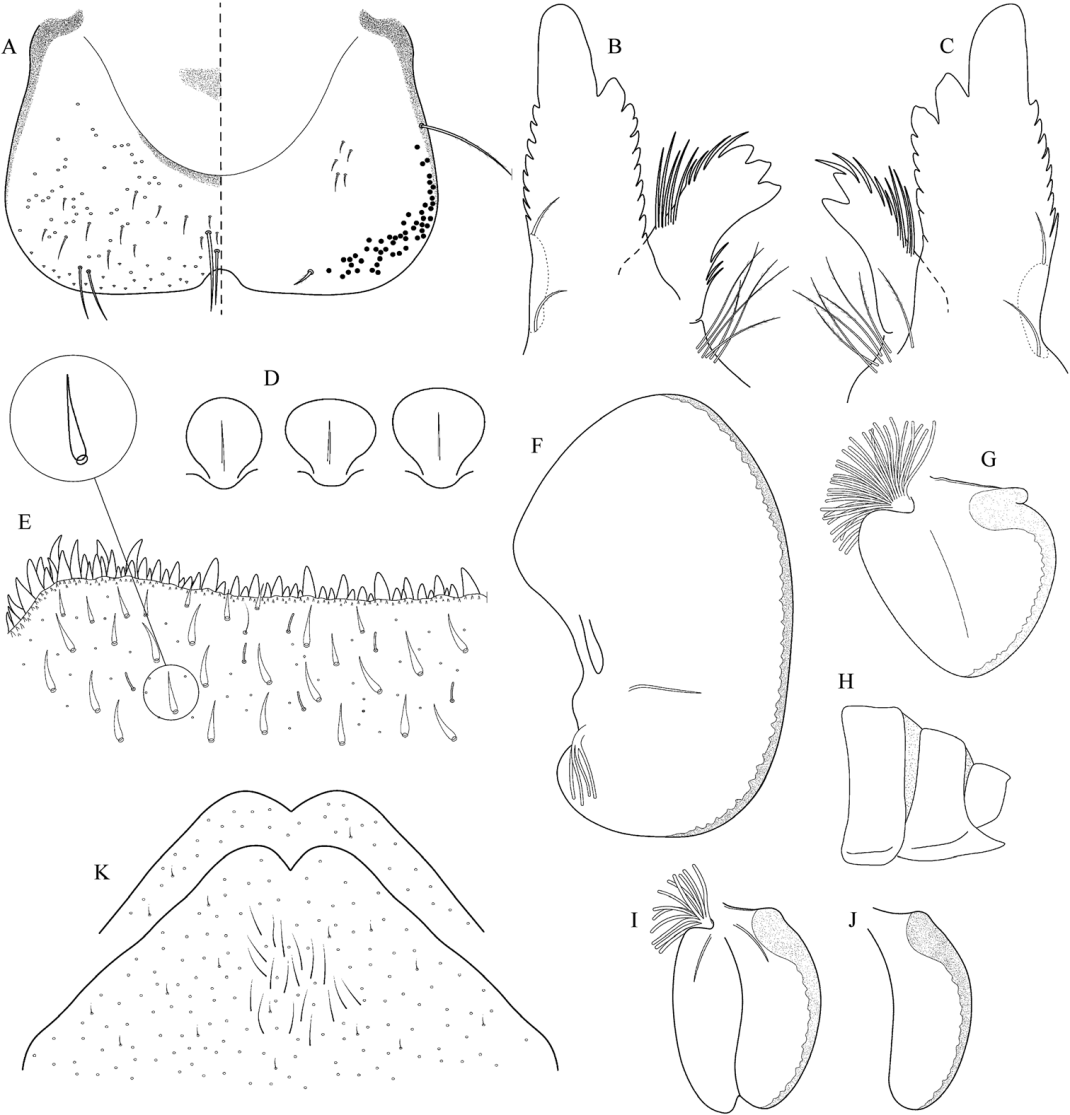
Epeorus (Caucasiron) bicolliculatus, larva: **A** labrum (left half in dorsal view right half in ventral view) **B** incisors of left mandible **C** incisors of right mandible **D** setae on dorsal surface of femora **E** surface and posterior margin of abdominal tergum VII with detail of basally wide setae **F** gill I **G** gill III **H** abdominal segments VIII–X in lateral view **I** gill VII (flattened on slide) **J** gill VII (in natural position from ventral view) **K** sternum IX of female with observed variability.

(i) abdominal terga II–IX with paired postero-medial protuberances (Fig. 34H, arrows); (ii) abdominal terga V–VII with stripe-like medial macula, often anteriorly and posteriorly widened, and with antero-lateral stripes (Fig. 34G, H); (iii) abdominal sterna as on Fig. 34B, J–L; (iv) setae on abdominal terga wide at base (Fig. 35E); (v) gill plates VII (in natural position from ventral view) narrow (Figs 34I, 35I, J); (vi) femora without medial hypodermal spot (Fig. 34F, blurred macula may be present in darker specimens); (vii) tergum X without postero-lateral projections (Fig. 35H); (viii) gill plates III with well-developed projection (Fig. 35G).

##### Remarks.

***Taxonomy*.** This species was described based on the larva, male subimago and imago (associated by rearing), female imago (associated by DNA analysis) and eggs. Material was collected from the western Lesser Caucasus (Hrivniak et al. 2017). The type series is currently deposited in IECA.

#### Epeorus (Caucasiron) turcicus

Taxon classificationAnimaliaEphemeropteraHeptageniidae

Hrivniak, Türkmen & Kazancı, 2019

04809F7B-F46F-51F1-8C2D-8DCA292A9AFF

Figs 36 , 37 , 38

##### Type locality.

Turkey, Artvin Province, Camili Village, Merata Plateau, unnamed mountain stream; 41°26'30"N/42°04'41"E; 2190 m a.s.l.

##### Distribution.

**Figure 36.**
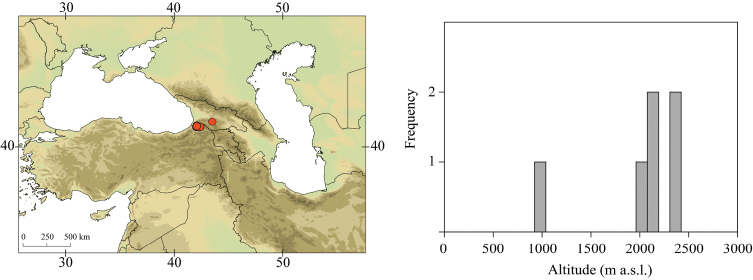
Geographical (left) and vertical (right) distribution of Epeorus (Caucasiron) turcicus.

North-eastern Turkey, Georgia (Fig. 36). Known only from few sites in the Camili (Machakheli) District in Turkey and central Georgia.

##### Habitat.

Larvae inhabit small streams at middle and high altitudes. Altitudinal range of sampling sites 928–2388 m a.s.l. (Fig. 36).

##### Main morphological diagnostics of larvae.

**Figure 37.**
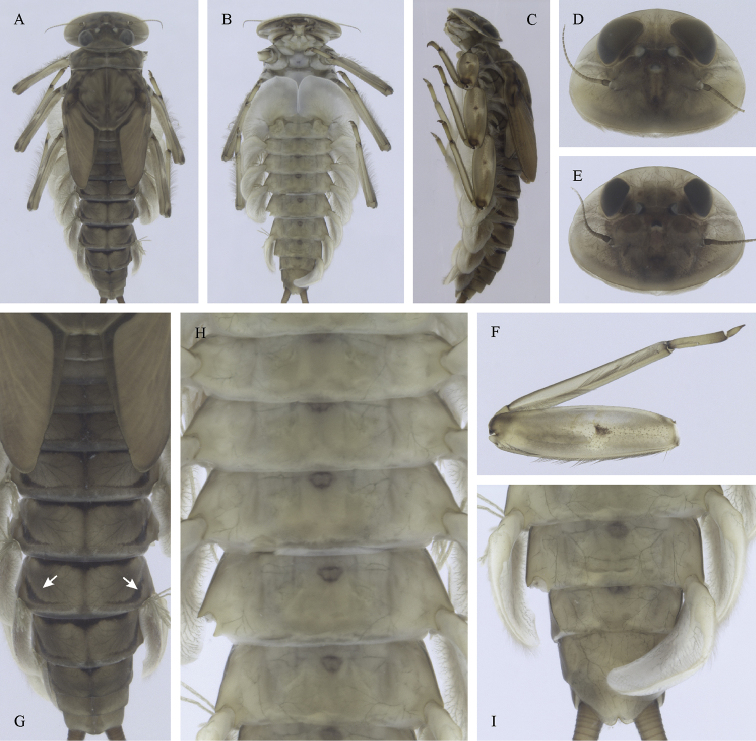
Epeorus (Caucasiron) turcicus, larva: **A** habitus in dorsal view **B** habitus in ventral view **C** habitus in lateral view **D** head of male in dorsal view **E** head of female in dorsal view **F** middle leg in dorsal view **G** abdominal terga (arrows point on dorso-posteriorly extended lateral stripes) **H** abdominal sterna II–VI **I** gill VII (in natural position from ventral view).

**Figure 38.**
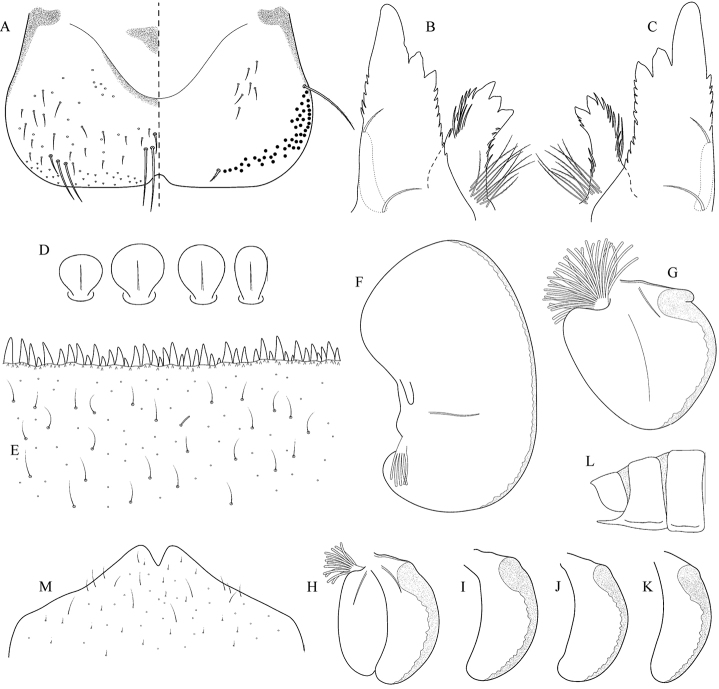
Epeorus (Caucasiron) turcicus, larva: **A** labrum (left half in dorsal view right half in ventral view) **B** incisors of left mandible **C** incisors of right mandible **D** setae on dorsal surface of femora **E** surface and posterior margin of abdominal tergum VII **F** gill I **G** gill III **H** gill VII (flattened on slide) **I–K** gill VII (in natural position from ventral view) variability in shape **L** abdominal segments VIII–X in lateral view **M** sternum IX of female.

(i) femora with medial hypodermal spot (Fig. 37F); (ii) abdominal terga V–VII with stripe-like medial macula with lateral stripes extended dorso-posteriorly (Fig. 37G, arrows); (iii) abdominal sterna without coloration pattern, nerve ganglia often coloured (Fig. 37B, H); (iv) gill plates VII (in natural position from ventral view) narrow (Figs 37I, 38H–K); (v) setae on abdominal terga hair-like like (Fig. 38E); (vi) tergum X without postero-lateral projections (Fig. 38L); (vii) gill plates III with well-developed projection (Fig. 38G).

##### Remarks.

***Taxonomy*.** This species described based on larvae collected from Pontic Mts. (Hrivniak et al. 2019). Imagines not described. The type series is currently deposited in IECA and collection of N. Kazancı and G. Türkmen (Hacettepe University, Department of Biology, Biomonitoring Laboratory, Turkey).

#### Epeorus (Caucasiron) alborzicus

Taxon classificationAnimaliaEphemeropteraHeptageniidae

Hrivniak & Sroka, 2020

DE1C9B3D-346B-5F01-BD8F-09A3713C35EA

Figs 39 , 40 , 41

##### Type locality.

Iran, Mazandaran Province, Panjab village, unnamed brook (left tributary of Haraz River); 36°05'52.818"N/52°15'15.987"E (locality no. 152); 955 m a.s.l.

##### Distribution.

**Figure 39.**
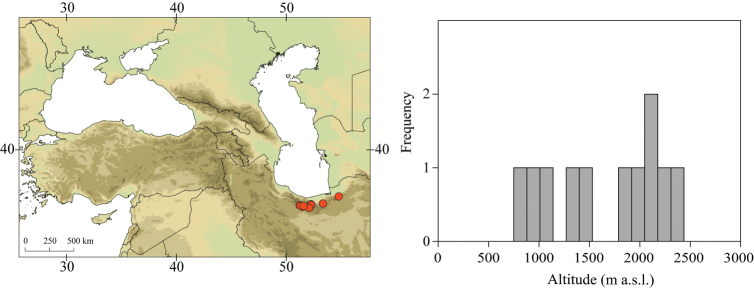
Geographical (left) and vertical (right) distribution of Epeorus (Caucasiron) alborzicus.

Northern Iran. Species endemic to the Alborz Mountains (Fig. 39).

##### Habitat.

Larvae inhabit small rivers at middle and high altitude in the central Alborz. Altitudinal range of sampling sites 750–2438 m a.s.l. (Fig. 39). Most frequently found at altitudes above 1000 m a.s.l. At high altitudes often syntopic with E. (C.) iranicus.

##### Main morphological diagnostics of larvae.

**Figure 40.**
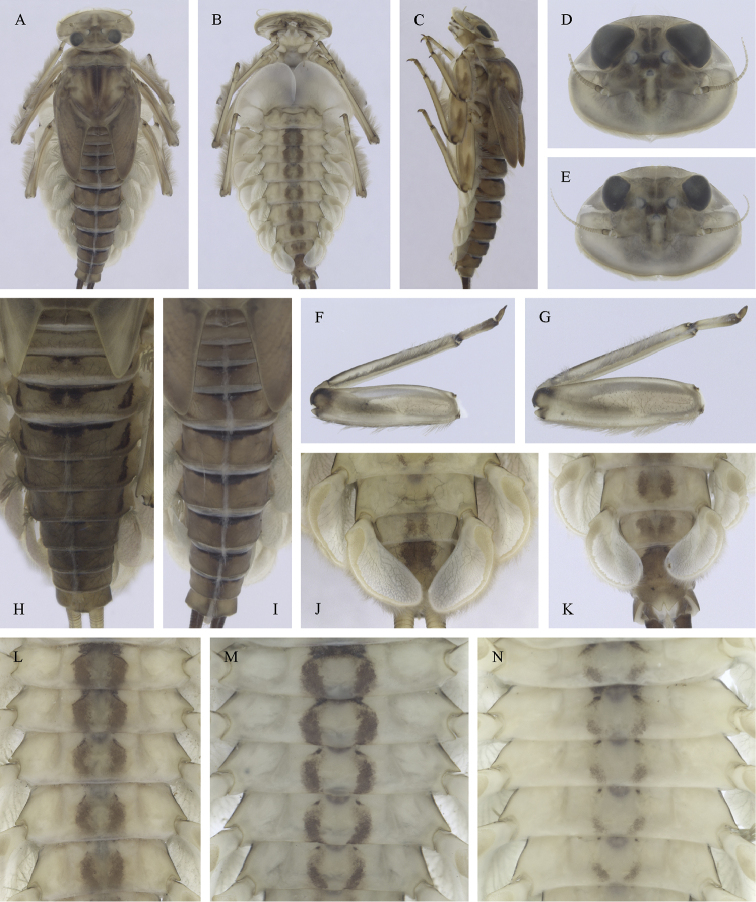
Epeorus (Caucasiron) alborzicus, larva: **A** habitus in dorsal view **B** habitus in ventral view **C** habitus in lateral view **D** head of male in dorsal view **E** head of female in dorsal view **F, G** middle leg in dorsal view **H, I** abdominal terga **J, K** gills VII (in natural position from ventral view) **L–N** abdominal sterna II–VI.

**Figure 41.**
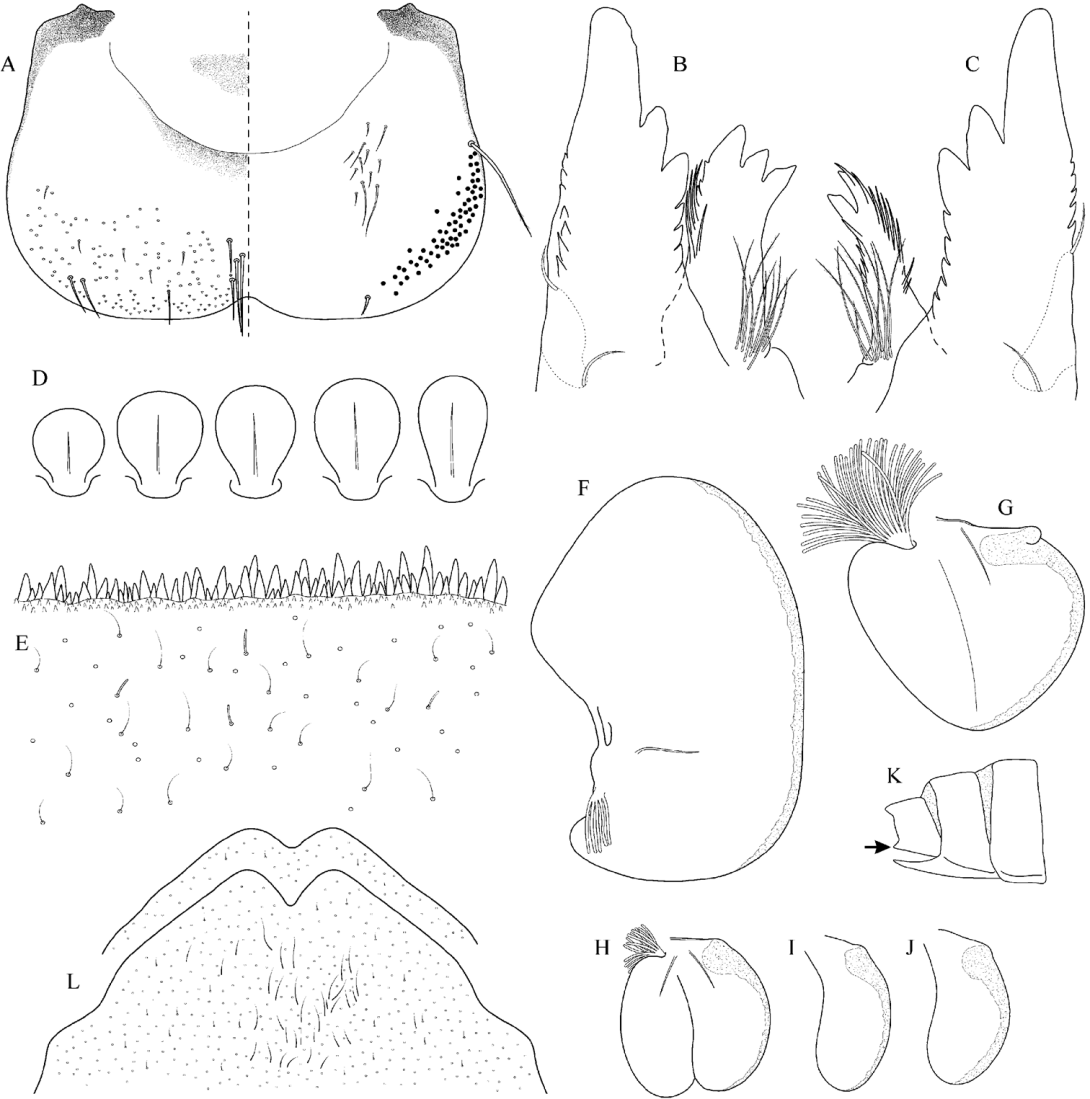
Epeorus (Caucasiron) alborzicus, larva: **A** labrum (left half in dorsal view right half in ventral view) **B** incisors of left mandible **C** incisors of right mandible **D** setae on dorsal surface of femora **E** surface and posterior margin of abdominal tergum VII **F** gill I **G** gill III **H** gill VII (flattened on slide) **I, J** gill VII (in natural position from ventral view) variability in shape **K** abdominal segments VIII–X in lateral view (arrow points on postero-lateral projection) **L** sternum IX of female with observed variability.

(i) abdominal terga as on Fig. 40H, I; (ii) abdominal sterna II–VI with circular central medial macula of various intensity (Fig. 40B, L–N); (iii) tergum X with postero-lateral projections (Fig. 41K, arrow), (iv) femora without medial hypodermal spot (Fig. 40F, G); (v) gill plates VII (in natural position from ventral view) wide (Figs 40J, K, 41H–J); (vi) setae on abdominal terga hair-like (Fig. 41E); (vii) gill plates III with well-developed projection (Fig. 41G).

##### Remarks.

***Taxonomy*.** This species was described based on larvae collected from Alborz Mts. (Hrivniak et al. 2020a). Imagines not described.

The type series is currently deposited in SMNS, IECA, and Natural History Museum and Genetic Resources, Department of Environment, Tehran, Iran (MMTT_DOE).

#### Epeorus (Caucasiron) shargi

Taxon classificationAnimaliaEphemeropteraHeptageniidae

Hrivniak & Sroka, 2020

FA6CD7B9-1957-5AE8-9962-21A1CB78E2E5

Figs 42 , 43 , 44

##### Type locality.

Iran, Golestan Province, Shirinabad village, unnamed river; 36°48'01.44"N/ 55°01'05.78"E (locality no. 108); 740 m a.s.l.

##### Distribution.

**Figure 42.**
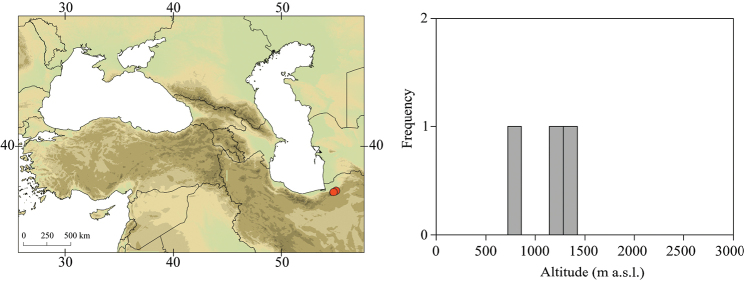
Geographical (left) and vertical (right) distribution of Epeorus (Caucasiron) shargi.

Northern Iran. Known only from three sites in the eastern Alborz (Fig. 42).

##### Habitat.

Larvae inhabit streams at middle altitude, 740–1450 m a.s.l. (Fig. 42).

##### Main morphological diagnostics of larvae.

**Figure 43.**
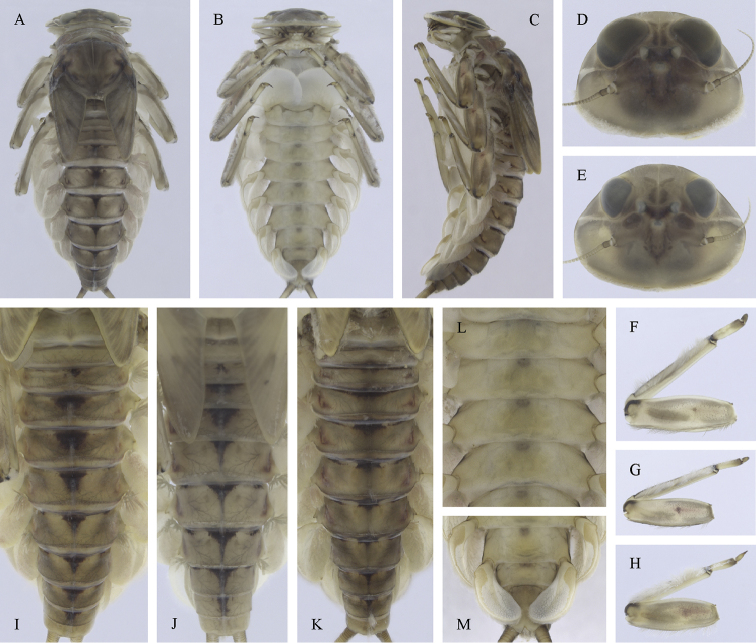
Epeorus (Caucasiron) shargi, larva: **A** habitus in dorsal view **B** habitus in ventral view **C** habitus in lateral view **D** head of male in dorsal view **E** head of female in dorsal view **F–H** middle leg in dorsal view **I–K** abdominal terga **L** abdominal sterna II–VI **M** gills VII (in natural position from ventral view).

**Figure 44.**
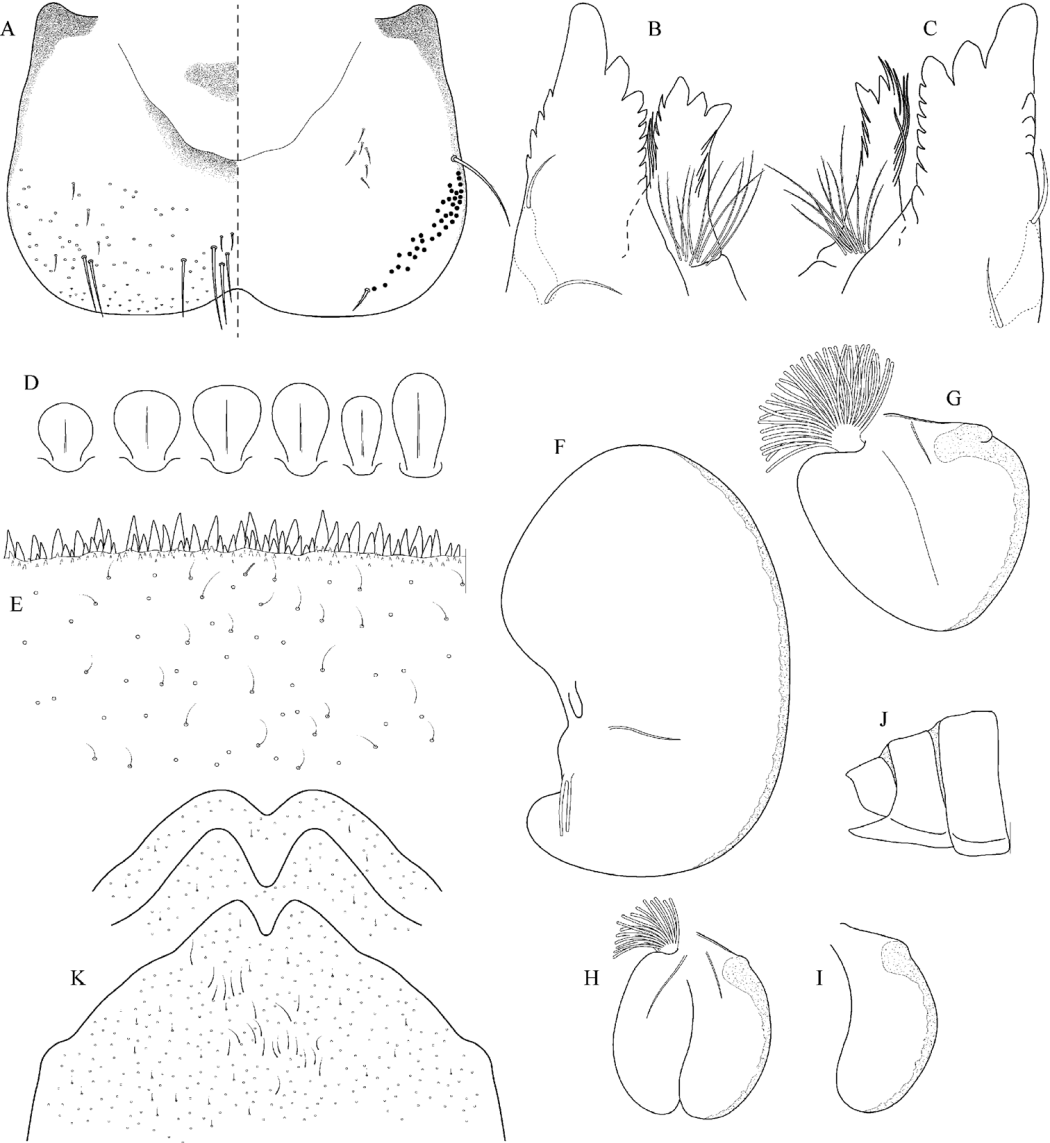
Epeorus (Caucasiron) shargi, larva: **A** labrum (left half in dorsal view right half in ventral view) **B** incisors of left mandible **C** incisors of right mandible **D** setae on dorsal surface of femora **E** surface and posterior margin of abdominal tergum VII **F** gill I **G** gill III **H** gill VII (flattened on slide) **I** gill VII (in natural position from ventral view) **J** abdominal segments VIII–X in lateral view **K** sternum IX of female with observed variability.

(i) abdominal terga V–VII with triangular or T-shaped medial macula (Fig. 43I–K); (ii) abdominal sterna without coloration pattern (Fig. 43B, L); (iii) tergum X without postero-lateral projections (Fig. 44J); (iv) femora with medial hypodermal spot (Fig. 43F–H); (v) gill plates VII (in natural position from ventral view) wide (Figs 43M, 44H, I); (vi) setae on abdominal terga hair-like (Fig. 44E); (vii) gill plates III with well-developed projection (Fig. 44G); (viii) shape of head of male oval trapezoidal (Fig. 43D).

##### Remarks.

***Taxonomy*.** This species was described based on larvae collected from Alborz Mts. (Hrivniak et al. 2020a). Imagines not described. The type series is currently deposited in SMNS, IECA, and MMTT_DOE.

#### Epeorus (Caucasiron) zagrosicus

Taxon classificationAnimaliaEphemeropteraHeptageniidae

Hrivniak & Sroka, 2020

21459487-E344-571A-BD4A-A70C25BCF288

Figs 45 , 46 , 47

##### Type locality.

Iran, Chaharmahal and Bakhtiari Province, Dimeh village, Chehme-Dimeh River, 32°30'11.62"N, 50°13'04.45"E; 2220 m a.s.l.

##### Distribution.

**Figure 45.**
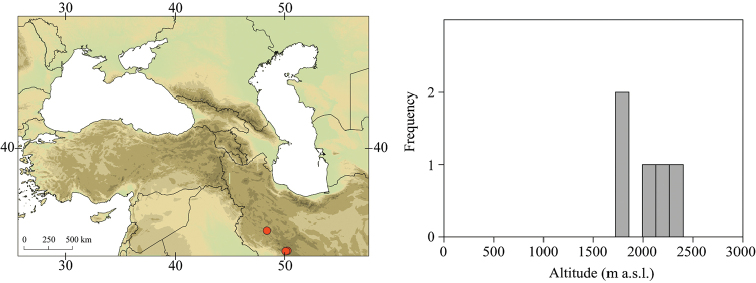
Geographical (left) and vertical (right) distribution of Epeorus (Caucasiron) zagrosicus.

South-western Iran. Known only from few sites in the central Zagros (Fig. 45).

##### Habitat.

Larvae inhabit streams and rivers at high altitude, 1721–2402 m a.s.l. (Fig. 45).

##### Main morphological diagnostics of larvae.

**Figure 46.**
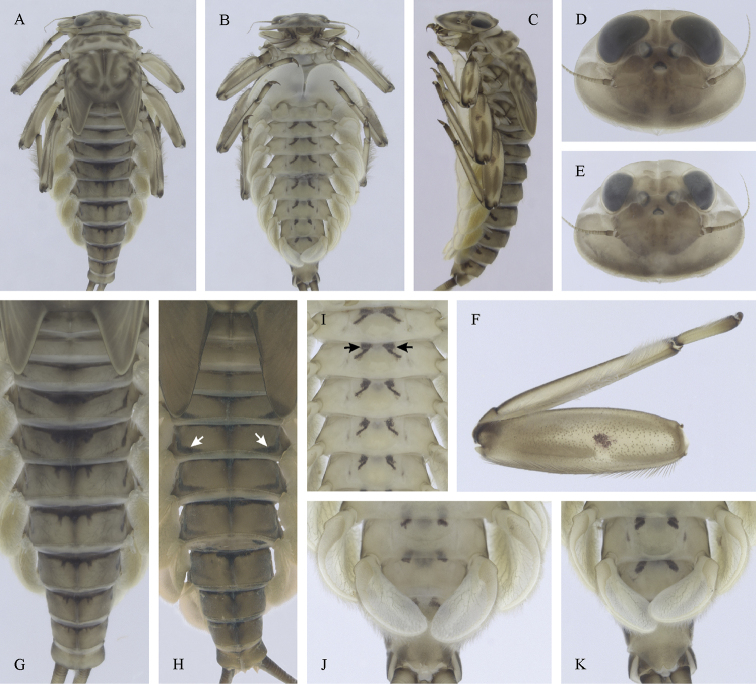
Epeorus (Caucasiron) zagrosicus, larva: **A** habitus in dorsal view **B** habitus in ventral view **C** habitus in lateral view **D** head of male in dorsal view **E** head of female in dorsal view **F** middle leg in dorsal view **G, H** abdominal terga (arrows point on dorso-posteriorly extended lateral stripes) **I** abdominal sterna II–VI **J, K** gills VII (in natural position from ventral view).

**Figure 47.**
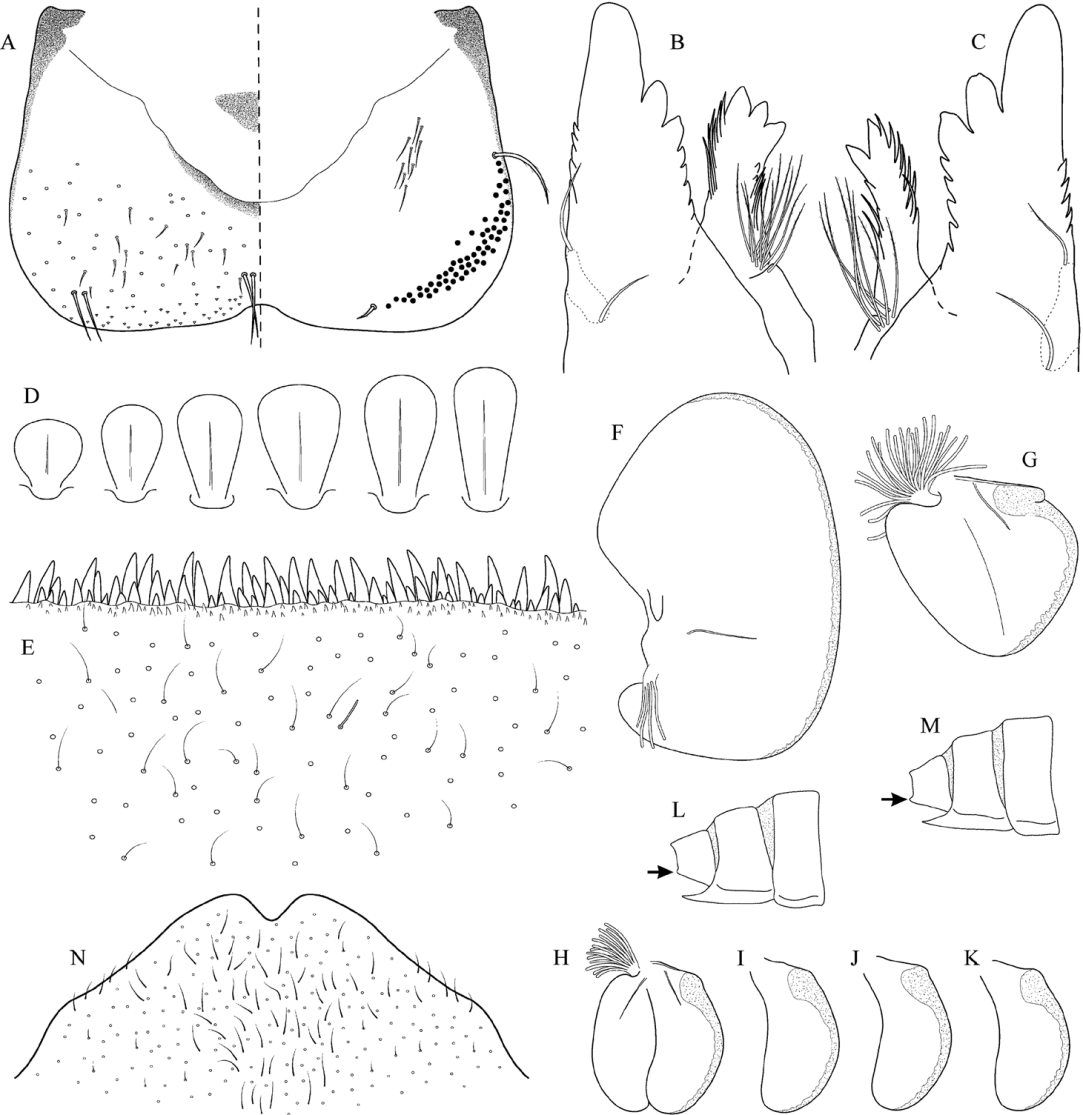
Epeorus (Caucasiron) zagrosicus, larva: **A** labrum (left half in dorsal view right half in ventral view) **B** incisors of left mandible **C** incisors of right mandible **D** setae on dorsal surface of femora **E** surface and posterior margin of abdominal tergum VII **F** gill I **G** gill III **H** gill VII (flattened on slide) **I–K** gill VII (in natural position from ventral view) variability in shape **L, M** abdominal segments VIII–X in lateral view (arrow point on postero-lateral projections) **N** sternum IX of female.

(i) abdominal sterna II–VI with a pair of anteriorly widened oblique stripes (Fig. 46B, I, arrows); (ii) abdominal terga V–VII with triangular, stripe-like or crown-like medial macula (Fig. 46G, H), often with lateral stripes extended dorso-posteriorly (Fig. 46H, arrows); (iii) tergum X with postero-lateral projections (Fig. 47L, M, arrows); (iv) femora with medial hypodermal spot (Fig. 46F); (v) setae on abdominal terga hair-like (Fig. 47E); (vi) gill plates III with well-developed projection (Fig. 47G); (vii) gill plates VII (in natural position from ventral view) relatively wide (Figs 46J, K, 47H–K).

##### Remarks.

***Taxonomy.***

This species was described based on larvae collected from Zagros Mts. (Hrivniak et al. 2020a). Imagines not described. The type series is currently deposited in SMNS, IECA, and MMTT_DOE. The lineage *Caucasiron* sp. 2 detected by Hrivniak et al. (2020b) is distributed in Turkey (Taurus Mts.) and morphologically corresponds to E. (C.) zagrosicus. Therefore, E. (C.) zagrosicus may represent a species complex.

## Concluding remarks

This contribution represents the first complete source of information for the routine identification of the larvae of all fifteen *Caucasiron* species occurring in the Caucasus and adjacent areas. It is possible that additional new *Caucasiron* species will be described from the region and some morphologically and genetically variable taxa, such as E. (C.) znojkoi, will be split into several species. This identification guide describes the state of the art at the time of publication.

All species of *Caucasiron* mayflies are charismatic animals, unique to the region. Some of them are endemic in a relatively limited area (especially for the Greater Caucasus and the Alborz Mts.) and may have considerable conservation value. We hope that this work will contribute to an increase in the knowledge of *Caucasiron* mayflies among hydrobiologists and ecologists. We would also like to encourage regional researchers to incorporate *Caucasiron* species as indicators in their biomonitoring surveys and water quality assessments.

## Supplementary Material

XML Treatment for Epeorus (Caucasiron) caucasicus

XML Treatment for Epeorus (Caucasiron) znojkoi

XML Treatment for Epeorus (Caucasiron) magnus

XML Treatment for Epeorus (Caucasiron) nigripilosus

XML Treatment for Epeorus (Caucasiron) alpestris

XML Treatment for Epeorus (Caucasiron) soldani

XML Treatment for Epeorus (Caucasiron) iranicus

XML Treatment for Epeorus (Caucasiron) sinitshenkovae

XML Treatment for Epeorus (Caucasiron) longimaculatus

XML Treatment for Epeorus (Caucasiron) insularis

XML Treatment for Epeorus (Caucasiron) bicolliculatus

XML Treatment for Epeorus (Caucasiron) turcicus

XML Treatment for Epeorus (Caucasiron) alborzicus

XML Treatment for Epeorus (Caucasiron) shargi

XML Treatment for Epeorus (Caucasiron) zagrosicus
